# Synthesis of the Tolerance-Inducing Oligosaccharide Lacto-*N*-Fucopentaose III Bearing an Activated Linker

**DOI:** 10.1002/open.201300024

**Published:** 2013-07-11

**Authors:** Junfeng Zhang, Lu Zou, Todd L Lowary

**Affiliations:** [a] Alberta Glycomics Centre and Department of Chemistry, University of Alberta, Gunning-Lemieux Chemistry CentreEdmonton, AB T6G 2G2 (Canada) E-mail: tlowary@ualberta.ca

**Keywords:** activated esters, immunomodulatory glycans, lacto-*N*-fucopentaose III (LNFPIII), regioselective glycosylation, oligosaccharides

## Abstract

A concise synthetic route to an immunomodulatory pentasaccharide, lacto-*N*-fucopentaose III (**1**) and its corresponding human serum albumin conjugate, is described. Key transformations of the strategy include two highly regio- and stereoselective glycosylations for the construction of disaccharide **10** and pentasaccharide **12**, a Birch reduction for deprotection of benzyl ethers, and a UV-promoted radical addition of a thiol to an alkene for modification of the aglycone.

## Introduction

Lacto-*N*-fucopentaose III (LNFPIII), a pentasaccharide containing the Lewis^X^ trisaccharide antigen, is an immunomodulatory glycan that is present on schistosome eggs.[1] The expression of LNFPIII on schistosome eggs has been shown to suppress host immune responses, which enables the parasite to escape detection of the mammalian host immune system thus facilitating survival.[1] LNFPIII is also found in breast milk and the urine of pregnant women, as well as the fetal brain, and has been speculated to have a similar protective immunomodulatory effect in the fetus.[1, 2] Recently, Burlingham and co-workers demonstrated that a LNFPIII conjugate can prolong allogeneic graft survival in neonatal heart transplantation models.[3] Although the biological role LNFPIII plays in graft prolongation remains to be further investigated, a preliminary mechanistic study suggested that the LNFPIII conjugate significantly upregulated the expression of programmed death ligand 1,[3] which negatively regulates immune responses through binding with its receptor, programmed cell death protein 1, expressed on the surface of activated T cells, B cells and macrophages.[4] This observation suggests that LNFPIII is a potential tolerance- inducing oligosaccharide.

**Figure 1:**
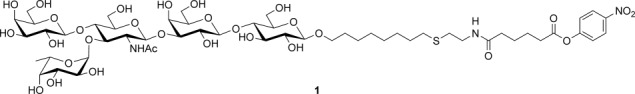
Structure of LNPFIII **1** with activated flexible linker.

We have been interested in accessing devices (nanoparticles or stents) carrying synthetic ABO blood group antigens and tolerance-inducing glycans for use in inducing specific B-cell tolerance during immune development, with the aim to extend the window for ABO-incompatible heart transplants.[5] As part of this program, we wanted to determine if LNFPIII presented together with the ABO blood group structures could promote immune tolerance to these antigens in neonates. Carrying out these studies required access to milligram quantities of LNPFIII functionalized with a linker that would allow its attachment to surfaces, for example, proteins as well as amine-coated nanoparticles or stents.[6] Although previous synthesis of LNPFIII derivatives have been reported by Sinaÿ[7] and Zhang,[8] none of these compounds was suitably functionalized for our purposes. We describe here the synthesis of LNPFIII bearing an activated ester moiety in the aglycone **1** (Figure 1) and its corresponding human serum albumin (HSA) conjugate.

## Results and Discussion

**Scheme 1:**
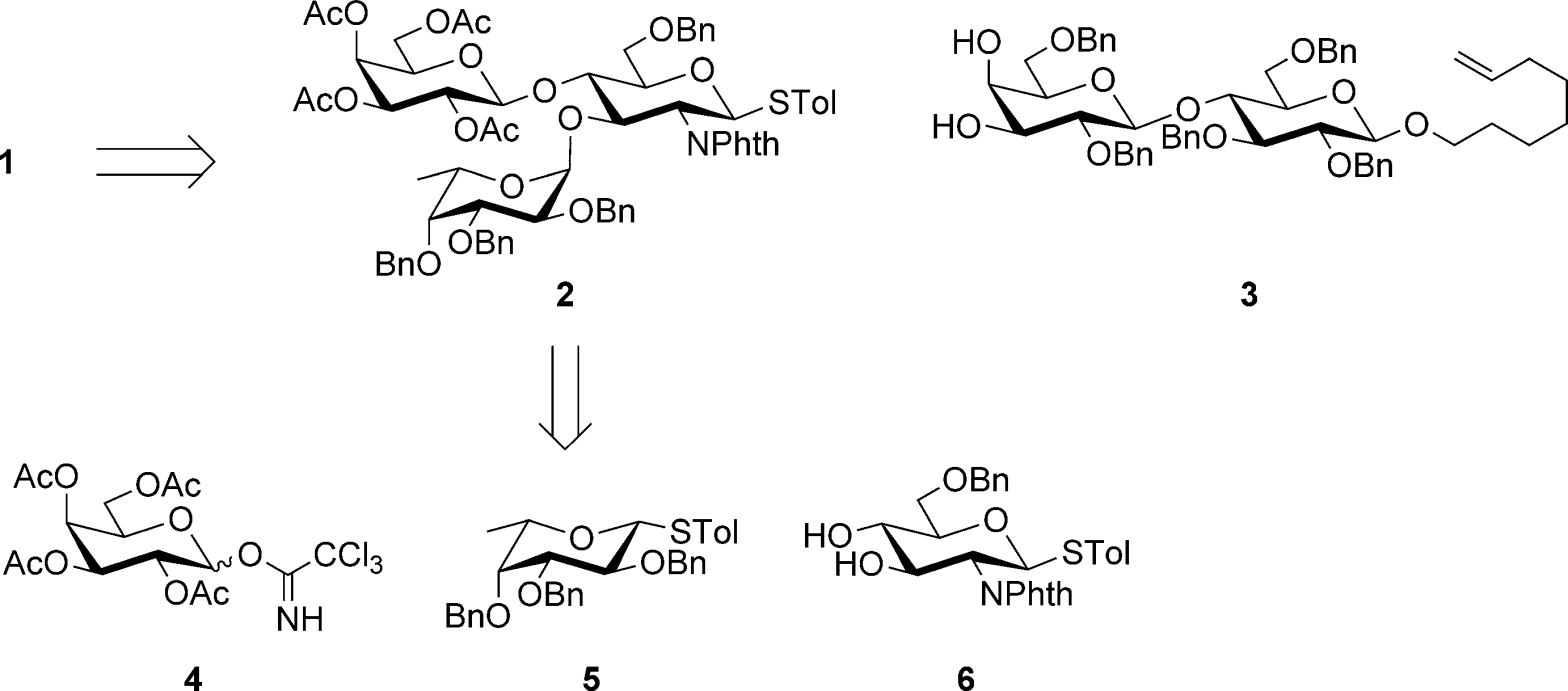
Retrosynthetic analysis of **1**. Bn=benzyl; Phth=phthaloyl; Tol=tolyl.

We envisioned (Scheme 1) constructing pentasaccharide **1** from four readily available carbohydrates, d-galactose, L-fucose, *N*-acetyl-d-glucosamine and lactose, via trisaccharide thioglycoside **2** and disaccharide diol **3**. An important feature was a regio- and stereoselective [3+2] glycosylation, a key strategy in earlier routes to LNPFIII derivatives,[7, 8] followed by global deprotection and introduction of the activated ester. Thioglycoside **2** can be assembled through regioselective condensation of trichloroacetimidate **4** with diol **6** followed by treatment of the product with thioglycoside **5**.

**Scheme 2:**
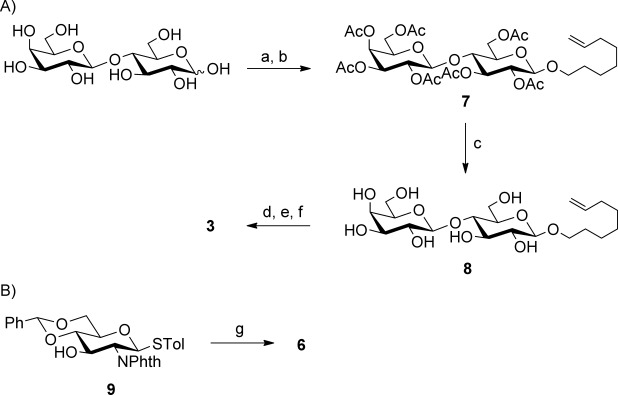
Synthesis of building blocks A) **3** and B) **6**. *Reagents and conditions*: a) Ac_2_O, NaOAc, 100 °C, 79 %; b) BF_3_⋅Et_2_O, 7-octen-1-ol, CH_2_Cl_2_, 43 %; c) NaOMe, 1:3 *v*/*v* CH_2_Cl_2_/MeOH, RT, overnight, 92 %; d) (CH_3_)_2_C(OCH_3_)_2_, *p*-TsOH, DMF, 85 °C, 1.5 h; e) NaH (6.6 equiv), BnBr (8.3 equiv), DMF, 0 °C→RT; f) 4:1 *v*/*v* AcOH/H_2_O, 80 °C, 3 h, 63 % over three steps; g) BH_3_⋅NMe_3_ (4.0 equiv), AlCl_3_ (6.0 equiv), THF, RT, 3 h, 83 %.

As illustrated in Scheme 2, diol **3** was prepared from lactose. First, acetylation was performed in acetic anhydride at 100 °C in the presence of sodium acetate to form preferentially the β anomer of lactose heptaacetate (α/β=1:5). This compound was then coupled with 7-octen-1-ol using boron trifluoride diethyl etherate as the promoter to generate octenyl glycoside **7** in 43 % overall yield. The ^1^H NMR spectrum of **7** showed the anomeric proton H-1 at 4.45 ppm as a doublet with a coupling constant between H-1 and H-2 of 8.0 Hz, indicating the newly formed glycosidic linkage was β. Then, deacetylation of **7** using a catalytic amount of sodium methoxide afforded **8** in 92 % yield. Finally, installation of an isopropylidene ketal at the 3′- and 4′-positions of **8**, benzylation of the remaining five hydroxyl groups, followed by acid hydrolysis of the isopropylidene ketal enabled the conversion of **8** to diol **3** in 63 % yield over three steps.[9] To access diol **6**, previously reported compound **9**[10] underwent regioselective reductive opening of the benzylidene ring using BH_3_⋅NMe_3_ and AlCl_3_[11] to the desired compound in 82 % yield. Building blocks **4**[12] and **5**[10] and were synthesized according to previous reports.

**Scheme 3:**
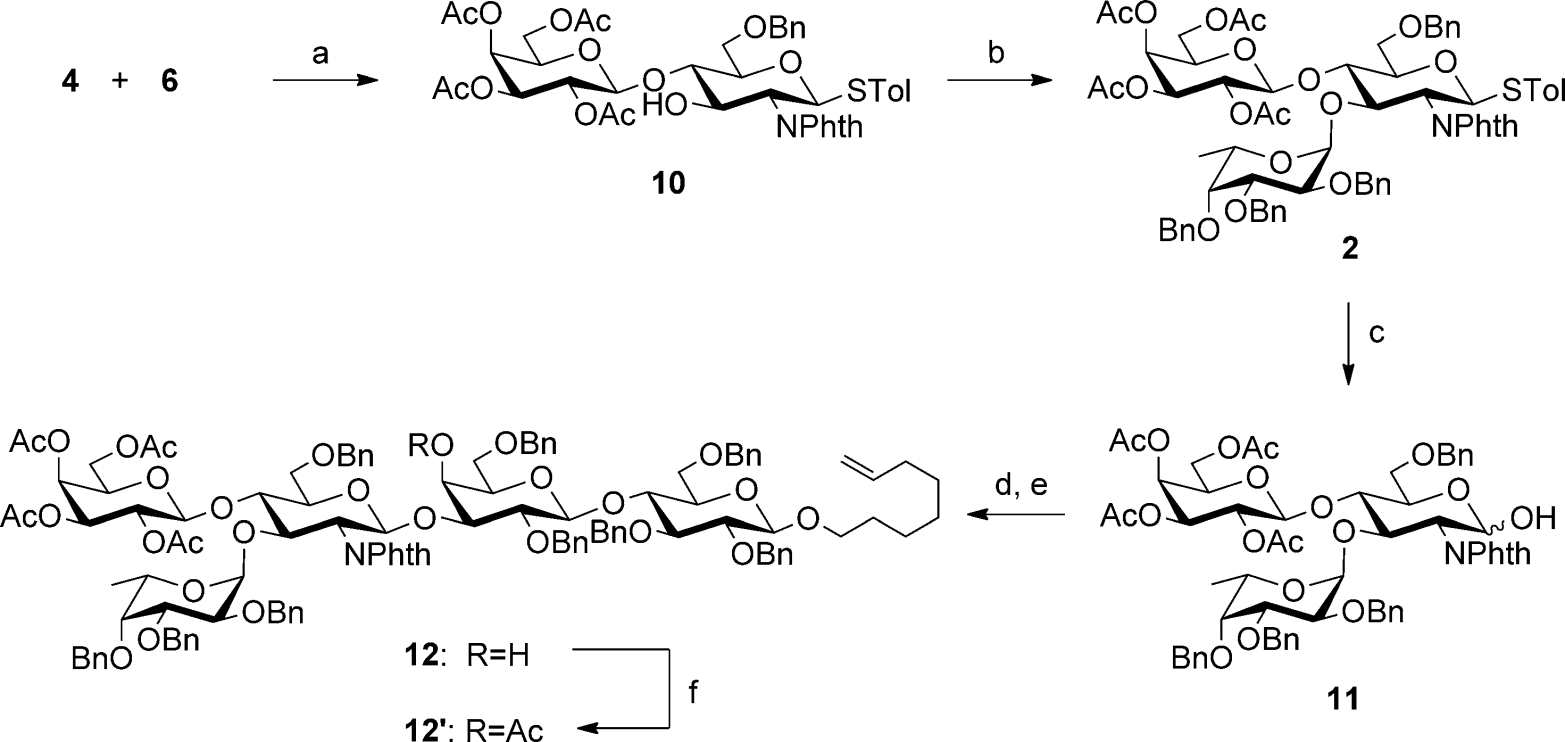
Synthesis of protected pentasaccharide **12**. *Reagents and conditions*: a) TMSOTf, CH_2_Cl_2_, −40 °C, 2 h 70 %; b) **5** (2.5 equiv), NIS (2.4 equiv), AgOTf (0.24 equiv), toluene, 0 °C, 75 %; c) NBS (2.5 equiv), 9:1 *v*/*v* acetone/H_2_O, 0 °C, 0.5 h, 80 %; d) DBU, Cl_3_CCN (7.0 equiv), CH_2_Cl_2_, RT, 4 h; e) **3** (1.3 equiv), TMSOTf, CH_2_Cl_2_, −20 °C, 49 % over two steps; f) Ac_2_O, pyridine, RT, overnight, 93 %.

With **3**–**6** in hand, the construction of the pentasaccharide was carried out (Scheme 3). First, trichloroacetimidate **4** was coupled with diol **6** in the presence of trimethylsilyl trifluoromethanesulfonate (TMSOTf) to generate regioselectively the β-(1→4)-linked disaccharide **10** in 70 % yield. The β stereochemistry was confirmed from the H-1, H-2 coupling constant of the galactopyranosyl residue (^3^*J*_H−1,H−2_=8.0 Hz). The regioselectivity of the glycosylation was determined by a 2D NMR experiment. In the HMBC spectrum of **10**, the expected correlations between H-1_Gal_ and C-4_GlcNAc_, H-4_GlcNAc_ and C-1_Gal_ are both observed, while correlations between H-1_Gal_ and C-3_GlcNAc_, H-3_GlcNAc_ and C-1_Gal_ are not, indicating that the newly formed glycosidic linkage is the desired β-(1→4)-linked disaccharide instead of the β-(1→3)-linked isomer. The regioselectivity of this glycosylation can be rationalized by matched–mismatched glycosylation.[13, 14] In this case, disarmed donor **4** reacts preferentially with the less reactive C-4 hydroxyl group in diol **6**. In addition, the steric effect induced by the *N*-phthalimido group at C-2 position also likely contributes to the regioselectivity.[13]

The fucosylation of disaccharide **10** with thioglycoside **5** in toluene using *N*-iodosuccinimide (NIS) and silver triflate (AgOTf) as the catalyst provided trisaccharide **2** in 75 % yield with excellent stereoselectivity. The anomeric proton of the fucopyranoside residue in **10** appeared around 4.84 ppm, overlapping with a methylene proton from the benzyl groups. Therefore, the one-bond heteronuclear coupling constant at the anomeric centre of the fucose residue (^1^*J*_C−1,H−1_)[15] was used to determine the stereochemistry at the newly formed linkage; the value is 170.3 Hz, which unambiguously confirms the α stereochemistry of this residue. The armed nature of **5** relative to **10**[10] allows this reaction to proceed efficiently without competing activation of the disaccharide acceptor or trisaccharide product.

The final planned glycosylation reaction, coupling of trisaccharide **2** and diol **3**, cannot be carried out using NIS and AgOTf activation because of incompatibility of these conditions with the alkene functionality in the aglycone. To circumvent this problem, an alternative promoter system using diphenylsulfoxide in combination with triflic anhydride[16] was explored, but the desired product was obtained in low yield. Although other thioglycoside activation conditions, for example using dimehylthiosulfonium triflate could have been explored, we chose instead to convert **2** into an alternate glycosyl donor. Therefore, thioglycoside **2** was treated with *N*-bromosuccinimide (NBS) in acetone/water (9:1 *v*/*v*) to afford hemiacetal **11** in 80 % yield. This compound was subsequently converted to corresponding trichloroacetimidate by treatment with trichloroacetonitrile in the presence of catalytic amount of 1,8-diazabicyclo[5.4.0]undec-7-ene (DBU). The freshly made donor was then used in a reaction with diol acceptor **3** to give the pentasaccharide **12** in 49 % yield over two steps. The β stereochemistry of the newly formed linkage was established based on the ^3^*J*_H−1, H−2_ (8.5 Hz) and ^1^*J*_C−1, H−1_ (165.5 Hz) values. To confirm the regioselectivity, a small amount of pentasaccharide **12** was acetylated to generate **12′**. Upon comparison of ^1^H NMR spectra of these two compounds, a broad signal at 4.05 ppm in **12** shifted to 5.46 ppm in **12’** and appeared as a doublet of doublets (*J*=3.6 and 0.6 Hz). The values of these coupling constants indicated that the acetyl group was introduced onto O-4**’** of the lactose moiety, confirming that the newly introduced glycosidic linkage was β-(1→3) not β-(1→4).

**Scheme 4:**
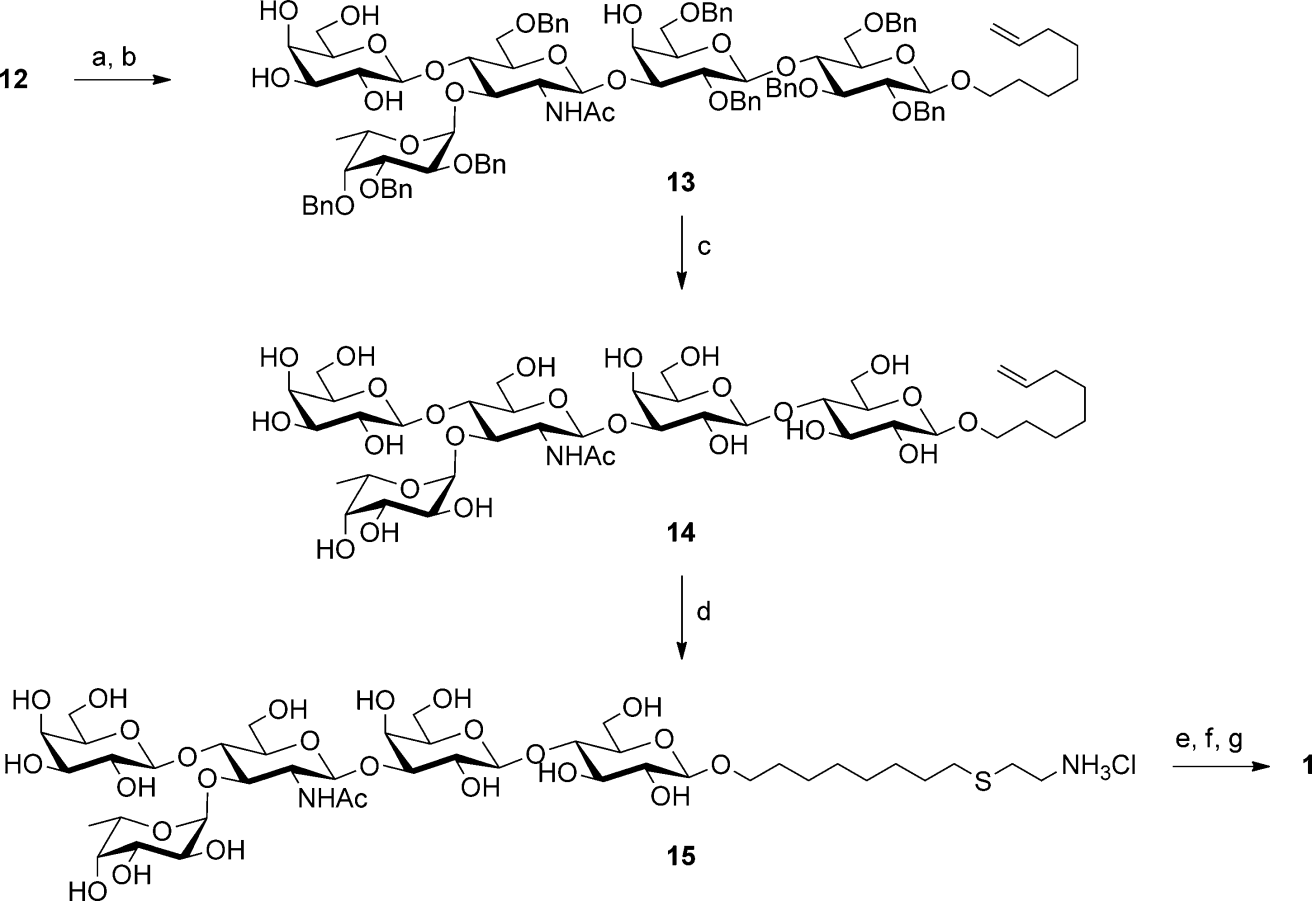
Synthesis of PNP ester **1**. *Reagents and conditions*: a) Ethylenediamine, *n*-butanol, 100 °C, 20 h; b) 1:2 *v*/*v* CH_2_Cl_2_/MeOH, Ac_2_O, Et_3_N, 83 % over two steps; c) Na, NH_3_, −78 °C, 2 h, 97 %; d) cysteamine–HCl (10.0 equiv), MeOH, UV, 2.5 h, quantitative, e) HO^−^ resin, MeOH, f) concentration, g) *N*,*N*-dimethylacetamide, di-*p*-nitrophenyl adipate (5.0 equiv), 83 %.

With the pentasaccharide assembled, treatment of **12** with ethylenediamine in *n*-butanol at 100 °C for 20 h (Scheme 4), followed by selective N-acetylation, led to formation of **13**. Birch reduction was conducted to remove the benzyl groups, while keeping intact the alkene functionality for further modification. The fully unprotected pentasaccharide **14** was obtained in 97 % yield. A UV-promoted radical addition of a thiol to the alkene[17] was then performed to further functionalize the octenyl linker with a cysteamine residue, leading to corresponding amine salt **15** in quantitative yield. Finally, the amine salt was converted to free amine by exchange with HO^**−**^ resin, followed by coupling with di-*p*-nitrophenyl adipate[18] in dimethylacetamide to yield the desired highly reactive *p*-nitrophenol (PNP) ester **1** in 83 % overall yield. Conversion of **1** into the corresponding human serum albumin (HSA) conjugate was then done by treatment of HSA with **1** in phosphate buffer (pH 7.5). The MALDI-MS spectrum of the resulting glycoconjugate showed two peaks, one centred around *m*/*z*=45 693 and another at *m*/*z*=91 377, corresponding to the +2 and +1 charge states of the protein, respectively, both bound to 21 pentasaccharide units.

## Conclusions

In conclusion, we have achieved the synthesis of LNPFIII functionalized with reactive *p*-nitrophenol ester (PNP) and the corresponding human serum albumin (HSA) conjugate. The synthetic strategy features two highly regio- and stereoselective glycosylations for the construction of disaccharide **10** and pentasaccharide **12** based on the reactivity difference between two hydroxyl groups in acceptor **6** and **3**, respectively. Birch reduction enabled the deprotection of benzyl ethers while leaving the octenyl linker intact, which allowed further functionalization to form PNP ester **1** and, in turn, the HSA conjugate. Studies on the use of this HSA conjugate in animal models of graft survival are ongoing.

## Experimental Section

**General**: All reagents were purchased from commercial sources and were used without further purification unless noted. Dry solvents used in reactions were purified by successive passage through columns of alumina and copper under an argon atmosphere. All reactions were carried out under a positive pressure of argon unless otherwise stated, monitored by TLC on silica gel 60 F_254_ (0.25 mm; Silicycle, Quebec, Canada), and the spots were visualized under UV light (254 nm) and/or stained by charring with acidified anisaldehyde solution in EtOH. Column chromatography was performed on silica gel 60 (40–60 μm) or C_18_ silica gel (35–70 μm, Toronto Research Chemicals). ^1^H NMR spectra were recorded at 500 or 600 MHz, and chemical shifts were referenced to CHCl_3_ (7.26 ppm, CDCl_3_), CD_2_HOD (3.31 ppm CD_3_OD), or HOD (4.79 ppm, D_2_O). ^13^C NMR spectra were recorded at 126 MHz and chemical shifts were referenced to CDCl_3_ (77.06 ppm, CDCl_3_), CD_3_OD (49.0 ppm, CD_3_OD) or external acetone (31.07 ppm, D_2_O). Assignments of NMR spectra were made on the basis of 2D experiments (^1^H–^1^H COSY, HSQC and HMBC), and the stereochemistry of the newly formed glycosidic linkages was confirmed by measuring ^1^*J*_C−1,H−1_ values using an ^1^H-coupled HSQC experiment. In the data provided below, the resonances on particular residues are indicated by an increasing number of primes (′) moving from the reducing to nonreducing end. For example, in **15** H-1 is H-1 of the Glc residue, H-1′′ is H-1 of the GlcNAc residue, and H-1′′′′ is H-1 of the Fuc residue. Electrospray ionization mass spectra were recorded on an Agilent Technologies 6220 TOF spectrometer on samples dissolved in CH_2_Cl_2_ or MeOH. MALDI mass spectra were obtained in the linear positive mode of ionization on a Bruker Daltonics (Bremen, GmbH) UltrafleXtreme MALDI TOF/TOF mass spectrometer using sinapinic acid as the matrix. Optical rotations were measured on PerkinElmer 241 polarimeter at 22±2 °C in units of degree mL/(g dm).

**6-[[2-[[8-(β-d-galactopyranosyl-(1→4)-[α-L-fucopyranosyl-(1→3)]-2-acetamido-2-deoxy-β-d-glucopyranosyl-(1→3)-β-d-galactopyranosyl-(1→4)-β-d-glucopyranosyl-oxy)octyl]thiol]ethyl]amino]-6-oxo-hexanoic acid**­ ***p*****-nitrophenyl ester (1)**: Compound **15** (10 mg, 9.3 μmol) was dissolved in MeOH (5 mL) and treated with Amberlite IR400 HO^**−**^ ion-exchange resin to convert the hydrochloride salt to the free amine. The solution was filtered, concentrated and dried overnight in vacuo. The residue was dissolved in *N*,*N*-dimethylacetamide (0.5 mL) and treated with di-*p*-nitrophenyl adipate (18 mg, 46.4 μmol). After stirring at RT for 4 h, the solution was concentrated in vacuo to dryness. The residue was subjected to C_18_ chromatography using gradient elution (0.5 % aq AcOH→ MeOH/0.5 % aq AcOH (60:40 *v*/*v*) to give **1** as a white foam after lyophilization (10 mg, 83 %): ^1^H NMR (600 MHz, CD_3_OD): *δ*=8.29 (d, *J*=9.2 Hz, 2 H, Ar), 7.37 (d, *J*=9.2 Hz, 2 H, Ar), 5.05 (d, *J*=4.0 Hz, 1 H, H-1′′′′), 4.83–4.82 (m, 1 H, H-5′′′′), 4.69 (d, *J*=8.1 Hz, 1 H, H-1′′), 4.43 (d, *J*=7.6 Hz, 1 H, one of H-1, H-1′, H-1′′′), 4.36 (d, *J*=7.7 Hz, 1 H, one of H-1, H-1′, H-1′′′), 4.27 (d, *J*=7.8 Hz, 1 H, one of H-1, H-1′, H-1′′′), 4.04 (d, *J*=3.0 Hz, 1 H), 3.95 (dd, *J*=6.3, 3.3 Hz, 1 H), 3.92–3.81 (m, 8 H), 3.79–3.75 (m, 3 H), 3.71–3.49 (m, 12 H), 3.46–3.41 (m, 3 H), 3.39–3.36 (m, 1 H), 3.35 (t, *J*=7.0 Hz, 2 H, SCH_2_C*H_2_*N), 3.22 (dd, *J*=9.1, 7.9 Hz, 1 H), 2.66 (t, *J*=7.1 Hz, 2 H, OC(O)C*H_2_*), 2.62 (t, *J*=7.0 Hz, 2 H, SC*H_2_*CH_2_N), 2.53 (t, *J*=7.3 Hz, 2 H, SC*H_2_*(CH_2_)_6_CH_2_O), 2.25 (t, *J*=7.0 Hz, 2 H, NHC(O)C*H_2_*), 1.97 (s, 3 H, NHAc), 1.77–1.73 (m, 4 H, NC(O)CH_2_(C*H_2_*)_2_CH_2_C(O)O), 1.61–1.55 (m, 4 H, OCH_2_C*H_2_*(CH_2_)_4_C*H_2_*CH_2_S), 1.39–1.30 (m, 8 H, OCH_2_CH_2_(C*H_2_*)_4_CH_2_CH_2_S), 1.17 ppm (d, *J*=6.6 Hz, 3 H, H-6′′′′); ^13^C NMR (126 MHz, CD_3_OD): *δ*=174.3 (C=O), 173.1 (C=O), 171.2 (C=O), 155.7 (Ar), 145.4 (Ar), 124.7 (2 C, Ar), 122.5 (2 C, Ar), 103.6, 102.8, 102.5 (C-1, C-1′, C-1′′′), 102.4 (C-1′′), 98.8 (C-1′′′′), 82.4, 79.1, 75.8, 75.3, 75.2, 75.1, 75.02, 74.99, 73.5 (2 C), 73.3, 72.3, 71.4, 70.1, 69.8, 69.5 (O*C*H_2_(CH_2_)_6_CH_2_S), 68.6 (2 C), 68.4, 66.3 (C-5′′′′), 61.4, 61.0, 60.5, 59.8 (C-6, C-6′, C-6′′, C-6′′′), 56.3 (C-2′′), 38.7 (SCH_2_*C*H_2_N), 35.2 (NHC(O)*C*H_2_), 33.2 (OC(O)*C*H_2_), 31.2 (S*C*H_2_CH_2_N), 30.8 (SC*H_2_*(CH_2_)_6_CH_2_O), 29.3 (2 C), 29.0, 28.8, 28.4 (5×SCH_2_(*C*H_2_)_6_CH_2_O), 25.6, 24.8, 23.8 (SCH_2_(*C*H_2_)_6_CH_2_O, 2×NC(O)CH_2_(*C*H_2_)_2_CH_2_C(O)O), 21.8 (NHAc), 15.2 ppm (C-6′′′′); HRMS (ESI): *m*/*z* [*M*+Na]^+^ calcd for C_54_H_87_N_3_NaO_30_S: 1312.4987, found: 1312.4986.

***p*-Tolyl 2,3,4–6-tetra-*O*-acetyl-β-d-galactopyranosyl-(1→4)-[2,3,4-tri-*O*-benzyl-α-L-fucopyranosyl-(1→3)]-6-*O*-benzyl-2-deoxy-2-phthalimido-1-thio-β-d-glucopyranoside (2)**: A mixture of thioglycoside **5** (2.74 g, 5.07 mmol), acceptor **10** (1.70 g, 2.03 mmol) and powdered 4 Å molecular sieves was suspended in toluene (30 mL) and stirred at RT for 1 h. The solution was cooled to 0 °C, and *N*-iodosuccinimide (1.10 g, 4.88 mmol) and silver trifluoromethanesulfonate (125 mg, 0.49 mmol) were added. After stirring at 0 °C for 2 h, Et_3_N (1 mL) was added, and the mixture was filtered through Celite. The filtrate was concentrated, and the resulting residue was subjected to flash chromatography (2:1 *v*/*v* hexane/EtOAc) to afford trisaccharide **2** as a white foam (1.90 g, 75 %): *R*_f_=0.24 (2:1 *v*/*v* hexane/EtOAc); [*α*]_D_=+7.1 (*c*=0.9, CHCl_3_); ^1^H NMR (500 MHz, CDCl_3_): *δ*=7.75–7.70 (m, 4 H, Ar), 7.45–7.01 (m, 24 H, Ar), 5.44 (d, *J*=10.5 Hz, 1 H, H-1), 5.26 (dd, *J*=3.5, 1.0 Hz, 1 H, H-4′), 5.04 (dd, *J*=10.4, 8.2 Hz, 1 H, H-2′), 4.84–4.79 (m, 4 H, H-1′′, H-3′, 2×OC*H*_2_Ph), 4.77–4.72 (m, 2 H, H-1′, H-3), 4.65–4.60 (m, 3 H, H-5′′, 2×OC*H*_2_Ph), 4.58 (A of ABq, *J*=11.7 Hz, 1 H, OC*H*_2_Ph), 4.53–4.48 (m, 2 H, H-2, OC*H*_2_Ph), 4.46, 4.28 (ABq, *J*=12.3 Hz, 2 H, 2×OC*H*_2_Ph), 4.18–4.13 (m, 2 H, H-4, H-6a′), 3.98 (dd, *J*=10.7, 5.7 Hz, 1 H, H-6b′), 3.92–3.88 (m, 2 H, H-3′′, H-6a), 3.85–3.80 (m, 2 H, H-2′′, H-6b) 3.64 (d, *J*=1.3 Hz, 1 H, H-4′′), 3.57–3.55 (m, 2 H, H-5, H-5′), 2.29 (s, 3 H, ArMe), 2.03 (s, 3 H, OAc), 2.02 (s, 3 H, OAc), 1.97 (s, 3 H, OAc), 1.86 (s, 3 H, OAc), 1.21 ppm (d, *J*=6.5 Hz, 3 H, H-6′′); ^13^C NMR (126 MHz, CDCl_3_): *δ* 170.0 (C=O), 169.8 (C=O), 168.7 (C=O), 138.9 (Ar), 138.7 (Ar), 138.2 (Ar), 138.1 (Ar), 137.9 (Ar), 134.2 (Ar), 133.3 (Ar), 131.8 (Ar), 129.6 (Ar), 128.6 (Ar), 128.3 (Ar), 128.24 (Ar), 128.17 (Ar), 128.1 (Ar), 128.02 (Ar), 127.97 (Ar), 127.86 (Ar), 127.4 (Ar), 127.2 (Ar), 127.1 (Ar), 127.0 (Ar), 123.7 (Ar), 99.5 (C-1′), 97.6 (C-1′′), 84.4 (C-1), 79.8 (C-3′′), 79.6 (C-5), 77.2 (C-4′′), 75.0 (C-4), 74.5 (C-2′′) 74.2 (O*C*H_2_Ph), 73.64 (C-3), 73.58, 73.0, 72.3 (3×O*C*H_2_Ph), 71.0 (C-3′), 70.4 (C-5′), 69.0 (C-2′), 67.85 (C-6), 66.76 (C-4′), 66.5 (C-5′′), 60.2 (C-6′), 55.6 (C-2), 21.1, 20.7, 20.62, 20.55, 20.54 ppm (4×OAc, ArMe); 16.7 (C-6′′); HRMS (ESI): *m*/*z* [*M*+Na]^+^ calcd for C_69_H_73_NNaO_19_S: 1274.4390, found: 1274.4385.

**7-Octen-1-yl 2,6-di-*O*-benzyl-β-d-galactopyranosyl-(1→4)-2,3,6-tri-*O*-benzyl-β-d-glucopyranoside (3)**: NaOMe (1.0 m, 2.7 mL) was added to a solution of **7** (2.89 g, 3.87 mmol) in CH_2_Cl_2_/MeOH (1:3 *v*/*v*, 40 mL). After stirring at RT overnight, the solution was neutralized with Amberlite IR120 H^+^ ion-exchange resin. The solution was filtered and the filtrate was concentrated to afford **8** as a white solid (1.61 g, 92 %). A portion of **8** (1.65 g, 3.65 mmol) and *p*-TsOH (41 mg) were suspended in a mixture of dry *N*,*N*-dimethylformamide (DMF; 8 mL) and 2,2-dimethoxypropane (24 mL). After stirring at 85 °C for 1.5 h, the solution was cooled to RT, neutralized with Et_3_N (1 mL), concentrated in vacuo and dried. The resulting residue and BnBr (2.9 mL, 24.3 mmol) were dissolved in dry DMF (40 mL), to which NaH (60 %, 1.22 g, 30.5 mmol) was added in portions at 0 °C, followed by vigorous stirring. After stirring overnight at RT, MeOH (4 mL) was added. The solution was concentrated in vacuo, dissolved in EtOAc (200 mL) and washed with H_2_O and brine. The organic layer was dried over Na_2_SO_4_, filtered and concentrated to dryness. Subsequently, the obtained crude residue was treated with 80 % aq HOAc (160 mL) at 80 °C for 3 h. The solution was concentrated, dissolved in EtOAc (200 mL) and washed with saturated aq NaHCO_3_, H_2_O and brine. The organic layer was dried over Na_2_SO_4_, filtered, concentrated and the residue subjected to flash chromatography (5:2 *v*/*v* hexane/EtOAc) to afford diol **3** as a white solid (2.07 g, 63 % over three steps): *R*_f_=0.40 (2:1 *v*/*v* hexane/EtOAc); [*α*]_D_=+16.4 (*c*=0.9, CHCl_3_); ^1^H NMR (500 MHz, CDCl_3_): *δ*=7.39–7.22 (m, 25 H, Ar), 5.81 (ddt, *J*=17.0, 10.3, 6.7 Hz, 1 H, C*H*=CH_2_), 5.02–4.92 (m, 3 H, CH=C*H*_2_, OC*H*_2_Ph), 4.91, 4.72 (ABq, *J*=11.0 Hz, 2 H, 2×OC*H*_2_Ph), 4.82, 4.68 (ABq, *J*=11.5 Hz, 2 H, 2×OC*H*_2_Ph), 4.79 (A of ABq, *J*=11.0 Hz, 1 H, OC*H*_2_Ph), 4.61 (A of ABq, *J*=12.5 Hz, 1 H, OC*H*_2_Ph), 4.47–4.44 (m, 3 H, H-1′, 2×OC*H*_2_Ph), 4.41–4.38 (m, 2 H, H-1, OC*H*_2_Ph), 3.99 (app t, *J*=9.5 Hz, 1 H, H-4), 3.97–3.92 (m, 2 H, H-4′, octenyl OCH_2_), 3.82 (dd, *J*=11.0, 4.0 Hz, 1 H, H-6a), 3.77 (dd, *J*=11.0, 2.0 Hz, 1 H, H-6b), 3.64–3.58 (m, 2 H, H-3, H-6a′), 3.54–3.49 (m, 2 H, H-6b′, octenyl OCH_2_), 3.45–3.39 (m, 4 H, H-2, H-2′, H-3′, H-5), 3.37–3.35 (m, 1 H, H-5′), 2.48 (d, *J*=3.5 Hz, 1 H, 4′-OH), 2.41 (d, *J*=4.0 Hz, 1 H, 3′-OH), 2.06–2.02 (m, 2 H, OCH_2_CH_2_(CH_2_)_3_C*H_2_*CH=CH_2_), 1.69–1.62 (m, 2 H, OCH_2_C*H_2_*(CH_2_)_3_C*H_2_*CH=CH_2_), 1.45–1.31 ppm (m, 6 H, OCH_2_CH_2_(C*H_2_*)_3_C*H_2_*CH=CH_2_); ^13^C NMR (126 MHz, CDCl_3_): *δ*=139.2 (Ar), 139.1 (*C*H=CH_2_), 138.7 (Ar), 138.4 (Ar), 138.3 (Ar), 138.0 (Ar), 128.5 (Ar), 128.4 (Ar), 128.32 (Ar), 128.30 (Ar), 128.08 (Ar), 128.06 (Ar), 128.0 (Ar), 127.93 (Ar), 127.86 (Ar), 127.7 (Ar), 127.6 (Ar), 127.57 (Ar), 127.54 (Ar), 127.3 (Ar), 114.3 (CH=*C*H_2_), 103.7 (C-1), 102.6 (C-1′), 82.9 (C-3), 81.8, 80.1 (C-2, C-2′), 76.7 (C-4), 75.2 (O*C*H_2_Ph), 75.1 (C-5), 74.93, 74.90 (2×O*C*H_2_Ph), 73.6 (C-3′), 73.5, 73.2 (2×O*C*H_2_Ph), 72.9 (C-5′), 70.0 (octenyl OCH_2_), 68.8 (C-4′), 68.7 (C-6′), 68.4 (C-6), 33.8, 29.7, 29.0, 28.9, 26.1 ppm (5×octenyl CH_2_); HRMS (ESI): *m*/*z* [*M*+Na]^+^ calcd for C_55_H_66_NaO_11_: 925.4497, found: 925.4489.

***p*****-Tolyl 6-*O*-benzyl-2-deoxy-2-phthalimido-1-thio-β-d-glucopyranoside (6)**: Benzylidene acetal **9** (1.82 g, 3.62 mmol) and BH_3_⋅NMe_3_ (1.06 g, 14.50 mmol) were dissolved in THF (26 mL) and cooled to 0 °C, then an ice-cold solution of AlCl_3_ (2.90 g, 21.74 mmol) in THF (10 mL) was added. After stirring at RT for 3 h, the solution was concentrated, dissolved in EtOAc (200 mL) and washed with saturated aq NaHCO_3_, H_2_O and brine. The organic layer was dried over Na_2_SO_4_, filtered, concentrated and subjected to flash chromatography (hexane/EtOAc 3:4 *v*/*v*) to afford diol **6** as a white solid (1.50 g, 82 %): *R*_f_=0.29 (3:4 *v*/*v* hexane/EtOAc); [*α*]_D_=+16.3 (*c*=1.0, CHCl_3_); ^1^H NMR (500 MHz; CDCl_3_): *δ*=7.87–7.85 (m, 2 H, Ar), 7.75–7.74 (m, 2 H, Ar), 7.40–7.29 (m, 7 H, Ar), 7.04–7.02 (m, 2 H, Ar), 5.56 (d, *J*=10.3 Hz, 1 H, H-1), 4.63, 4.58 (ABq, *J*=11.4 Hz, 2 H, 2×OC*H*_2_Ph), 4.35 (dd, *J*=10.3, 8.3 Hz, 1 H, H-3), 4.21 (app t, *J*=10.3 Hz, 1 H, H-2), 3.85 (dd, *J*=10.5, 4.5 Hz, 1 H, H-6a), 3.81 (dd, *J*=10.5, 4.5 Hz, 1 H, H-6b), 3.68–3.60 (m, 2 H, H-4, H-5), 2.30 ppm (s, 3 H, OAc); ^13^C NMR (126 MHz; CDCl_3_): *δ*=138.2 (Ar), 137.7 (Ar), 134.2 (Ar), 133.3 (Ar), 131.7 (Ar), 129.6 (Ar), 128.5 (Ar), 128.2 (Ar), 127.9 (Ar), 127.8 (Ar), 83.8 (C-1), 77.8 (C-5), 73.8 (OC*H*_2_Ph), 73.6 (C-4), 72.8 (C-3), 70.5 (C-6), 55.4 (C-2), 21.1 ppm (ArMe); HRMS (ESI): *m*/*z* [*M*+Na]^+^ calcd for C_28_H_27_NNaO_6_S: 528.1451, found: 528.1451.

**7-Octen-1-yl 2,3,4,6-tetra-*O*-acetyl-β-d-galactopyranosyl-(1→4)-2,3,6-tri-*O*-acetyl-β-d-glucopyranoside (7)**: d-Lactose (10.00 g, 29.2 mmol) and NaOAc (2.5 g, 30.5 mmol) were heated in Ac_2_O (80 mL) at 100 °C for 5 h. The solution was allowed to cool to RT and poured into ice water (1 L). The precipitate was filtered and recrystallized from EtOAc/hexane to yield peracetylated lactose as colourless crystals (15.60 g, 79 %). A portion of this compound (6.78 g, 10 mmol), 7-octen-1-ol (2.4 mL, 16 mmol) and 4 Å molecular sieves was dissolved in CH_2_Cl_2_ (80 mL) and stirred at RT for 0.5 h. The solution was cooled to 0 °C, and BF_3_⋅Et_2_O (3.8 mL, 30 mmol) was added. After stirring at RT for 24 h, Et_3_N (5 mL) was added, and the reaction mixture was filtered through Celite. The filtrate was concentrated and the residue was subjected to flash chromatography (4:3 *v*/*v* hexane/EtOAc) to afford **7** as white foam (3.2 g, 43 %): *R*_f_=0.39 (1:1 *v*/*v* hexane/EtOAc); [*α*]_D_=−11.6 (*c*=1.1, CHCl_3_); ^1^H NMR (500 MHz, CDCl_3_): *δ*=5.79 (ddt, *J*=17.0, 10.3, 6.7 Hz, 1 H, C*H*=CH_2_), 5.34 (dd, *J*=3.4, 1.1 Hz, 1 H, H-4′), 5.19 (app t, *J*=9.5 Hz, 1 H, H-3), 5.10 (dd, *J*=10.4, 7.9 Hz, 1 H, H-2′), 5.01–4.91 (m, 3 H, CH=C*H_2_*, H-3′), 4.88 (dd, *J*=9.5, 8.0 Hz, 1 H, H-2), 4.49–4.46 (m, 2 H, H-1′, H-6a), 4.45 (d, *J*=8.0 Hz, 1 H, H-1), 4.15–4.06 (m, 3 H, H-6b, H-6a′, H-6b′), 3.88–3.85 (m, 1 H, H-5′), 3.82 (dt, *J*=9.7, 6.8 Hz, 1 H, octenyl OCH_2_), 3.79 (app t, *J*=9.5 Hz, 1 H, H-4), 3.59 (ddd, *J*=9.9, 5.1, 2.1 Hz, 1 H, H-5), 3.44 (dt, *J*=9.7, 6.8 Hz, 1 H, octenyl OCH_2_), 2.15 (s, 3 H, OAc), 2.11 (s, 3 H, OAc), 2.06–2.03 (m, 14 H, 4×OAc, OCH_2_CH_2_(CH_2_)_3_C*H_2_*CH=CH_2_), 1.96 (s, 3 H, OAc), 1.57–1.51 (m, 2 H, OCH_2_C*H_2_*(CH_2_)_3_CH_2_CH=CH_2_), 1.39–1.25 ppm (m, 6 H, OCH_2_CH_2_(C*H_2_*)_3_CH_2_CH=CH_2_); ^13^C NMR (126 MHz, CDCl_3_): *δ*=170.4 (C=O), 170.3 (C=O), 170.1 (C=O), 170.0 (C=O), 169.8 (C=O), 169.6 (C=O), 169.1 (C=O), 139.0 (*C*H=CH_2_), 114.3 (CH=*C*H_2_), 101.1 (C-1′), 100.6 (C-1), 76.4 (C-4), 72.9 (C-3), 72.6 (C-5), 71.8 (C-2), 71.0 (C-3′), 70.7 (C-5′), 70.2 (octenyl OCH_2_), 69.1 (C-2′), 66.6 (C-4′), 62.1 (C-6), 60.8 (C-6′), 33.7, 29.4, 28.84, 28.77, 25.7 (5×octenyl CH_2_); 20.9, 20.8, 20.71, 20.66, 20.65 (2C), 20.5 ppm (7×OAc); HRMS (ESI): *m*/*z* [*M*+Na]^+^ calcd for C_34_H_50_NaO_18_: 769.2889, found: 769.2880.

***p*-Tolyl 2,3,4–6-tetra-*O*-acetyl-β-d-galactopyranosyl-(1→4)-6-*O*-benzyl-2-deoxy-2-phthalimido-1-thiol-β-d-glucopyranoside (10)**: A mixture of trichloroacetimidate **4** (1.80 g, 3.65 mmol), diol **6** (1.76 g 3.48 mmol) and powdered 4 Å molecular sieves was suspended in CH_2_Cl_2_ (40 mL) and stirred at RT for 1 h. The solution was cooled to −40 °C, and trimethylsilyl trifluoromethanesulfonate (TMSOTf; 63 μL) was added drop wise. After stirring at −40 °C for 2 h, the mixture was allowed to warm to RT. Et_3_N (1 mL) was added and the mixture was filtered through Celite. The filtrate was concentrated and subjected to flash chromatography (4:3 *v*/*v* hexane/EtOAc) to afford **10** as a white foam (2.21 g, 70 %): *R*_f_=0.39 (1:1 *v*/*v* hexane/EtOAc); [*α*]_D_=+18.5 (*c*=0.9, CHCl_3_); ^1^H NMR (500 MHz, CDCl_3_): *δ*=7.88–7.82 (m, 2 H, Ar), 7.75–7.71 (m, 2 H, Ar), 7.41–7.37 (m, 2 H, Ar), 7.36–7.30 (m, 5 H, Ar), 7.02–6.99 (m, 2 H, Ar), 5.53 (d, *J*=10.5 Hz, 1 H, H-1), 5.31 (dd, *J*=3.5, 1.0 Hz, 1 H, H-4′), 5.18 (dd, *J*=10.5, 8.0 Hz, 1 H, H-2′), 4.93 (dd, *J*=10.5, 3.5 Hz, 1 H, H-3′), 4.68, 4.52 (ABq, *J*=12.0 Hz, 2 H, 2×OC*H*_2_Ph), 4.50 (d, *J*=8.0 Hz, 1 H, H-1′), 4.39 (dd, *J*=10.5, 8.0 Hz, 1 H, H-3), 4.20 (app t, *J*=10.5 Hz, 1 H, H-2), 4.05–4.00 (m, 3 H, H-6a′, H-6b′, 3-OH), 3.89 (dt, *J*=6.5, 1.0 Hz, 1 H, H-5′), 3.76–3.64 (m, 4 H, H-4, H-5, H-6a, H-6b), 2.27 (s, 3 H, ArMe), 2.11 (s, 3 H, OAc), 2.00 (s, 3 H, OAc), 1.97 (s, 3 H, OAc), 1.89 ppm (s, 3 H, OAc); ^13^C NMR (126 MHz, CDCl_3_): *δ*=170.4 (C=O), 170.1 (C=O), 169.9 (C=O), 169.2 (C=O), 168.2 (C=O), 167.5 (C=O), 138.3 (Ar), 138.2 (Ar), 134.1 (Ar), 133.7 (Ar), 131.9 (Ar), 131.8 (Ar), 129.6 (Ar), 128.5 (Ar), 127.84 (Ar), 127.81 (Ar), 123.6 (Ar), 123.3 (Ar), 101.6 (C-1′), 83.4 (C-1), 81.8 (C-4), 78.2 (C-5), 73.7 (O*C*H_2_Ph), 71.2 (C-5′), 70.87 (C-3), 70.78 (C-3′), 68.7 (C-2′), 68.2 (C-6), 66.8 (C-4′), 61.4 (C-6′), 55.2 (C-2), 21.1, 20.7, 20.6, 20.5, 20.3 ppm (5C, 4×OAc, ArMe); HRMS (ESI): *m*/*z* [M+Na]^+^ calcd for C_42_H_45_NNaO_15_S: 858.2402, found: 858.2395.

**7-Octen-1-yl 2,3,4–6-tetra-*O*-acetyl-β-d-galactopyranosyl-(1→4)-[2,3,4-tri-*O*-benzyl-α-L-fucopyranosyl-(1→3)]-6-*O*-benzyl-2-deoxy-2-phthalimido-β-d-glucopyranosyl-(1→3)-2,6-di-*O*-benzyl-β-d-galactopyranosyl-(1→4)-2,3,6-tri-*O*-benzyl-β-d-glucopyranoside (12)**: *N*-Bromosuccinimide (NBS; 287 mg, 1.62 mmol) was added to a solution of trisaccharide **2** (809 mg, 0.65 mmol) in acetone/H_2_O (9:1 *v*/*v*, 7 mL) at 0 °C. After stirring at 0 °C for 0.5 h, saturated aq NaHCO_3_ (2 mL) was added. The solution was concentrated, and the residue was dissolved in EtOAc (80 mL) and washed with H_2_O and brine. The organic layer was dried over Na_2_SO_4_, filtered, concentrated and subjected to flash chromatography (4:5 *v*/*v* hexane/EtOAc) to afford **11** as a white solid (590 mg, 80 %). A solution of **11** (455 mg, 0.39 mmol) in CH_2_Cl_2_ (4 mL) was treated with trichloroacetonitrile (0.28 mL, 2.75 mmol) and catalytic amount of 1,8-diazabicyclo[5.4.0]undec-7-ene (DBU), and stirred at RT for 4 h. Concentration and flash chromatography (1:1 *v*/*v* hexanes/EtOAc) afforded the trichloroacetimidate that was immediately used in the next step. The trichloroacetimidate (387 mg, 0.3 mmol), diol **3** (354 mg, 0.39 mmol) and powdered 4 Å molecular sieves were suspended in CH_2_Cl_2_ (4 mL) and stirred at RT for 1 h. The solution was then cooled to −30 °C, to which TMSOTf (14 μL) was added. The mixture was allowed to warm to RT, and after stirring at −30 °C for 2 h, Et_3_N (1 mL) was added, and the mixture was filtered through Celite. The filtrate was concentrated and subjected to flash chromatography (5:2 *v*/*v* hexane/EtOAc) to afford **12** as a white foam (390 mg, 49 % over two steps): *R*_f_=0.46 (5:3 *v*/*v* hexane/EtOAc); [*α*]_D_=+5.5 (*c*=1.0, CHCl_3_); ^1^H NMR (600 MHz, CDCl_3_): *δ*=7.42–7.07 (m, 45 H, Ar), 7.01 (d, *J*=7.2 Hz, 2 H, Ar), 6.79 (d, *J*=7.2 Hz, 2 H, Ar), 5.78 (ddt, *J*=17.0, 10.3, 6.7 Hz, 1 H, C*H*=CH_2_), 5.32 (d, *J*=8.5 Hz, 1 H, H-1′′), 5.25 (dd, *J*=3.5, 0.8 Hz, 1 H, H-4′′′), 5.03 (dd, *J*=10.4, 8.2 Hz, 1 H, H-2′′′), 4.97 (dq, *J*=17.1, 1.8 Hz, 1 H, CH=C*H_2_*), 4.93–4.89 (m, 2 H, CH=C*H_2_*, OC*H*_2_Ph), 4.84–4.78 (m, 4 H, H-1′′′′, H-3′′′, 2 × OC*H*_2_Ph), 4.74–4.65 (m, 5 H, H-1′′′, H-3′′, 3×OC*H*_2_Ph), 4.60–4.53 (m, 4 H, H-5′′′′, 3×OC*H*_2_Ph), 4.48–4.42 (m, 4 H, H-2′′, 3×OC*H*_2_Ph), 4.33 (A of ABq, *J*=12.2 Hz, 2 H, 2×OC*H*_2_Ph), 4.30–4.25 (m, 2 H, H-1′, OC*H*_2_Ph), 4.21–4.17 (m, 3 H, H-1, 2×OC*H*_2_Ph), 4.16–4.12 (m, 3 H, H-4′′, H-6a′′′, OC*H*_2_Ph), 4.05 (br s, 1 H, H-4′), 3.95 (dd, *J*=10.9, 5.9 Hz, 1 H, H-6b′′′), 3.85–3.81 (m, 4 H, H-3′′′′, H-4, H-6a′′, octenyl OCH_2_), 3.75 (dd, *J*=12.2 Hz, 1 H, H-2′′′′), 3.71–3.68 (m, 2 H, H-6b′′, H-6a′), 3.61–3.58 (m, 3 H, H-4′′′′, H-5′′, H-5′′′), 3.49 (dd, *J*=9.6, 5.6 Hz, 1 H, H-6b′), 3.42 (dd, *J*=10.8 Hz, 4.2 Hz, 1 H, H-6a), 3.42–3.35 (m, 5 H, H-2′, H-3′, H-3, H-5′, octenyl OCH_2_), 3.32–3.27 (m, 2 H, H-2, H-6b), 2.96 (ddd, *J*=9.6, 4.2, 1.8 Hz, 1 H, H-5), 2.71 (br s, 1 H, 4′-OH), 2.03–1.99 (m, 8 H, 2×OAc, OCH_2_CH_2_(CH_2_)_3_C*H_2_*CH=CH_2_), 1.96 (s, 3 H, OAc), 1.85 (s, 3 H, OAc), 1.62–1.55 (m, 2 H, OCH_2_*CH_2_*(CH_2_)_3_CH_2_CH=CH_2_), 1.37–1.26 (m, 6 H, OCH_2_CH_2_(C*H_2_*)_3_CH_2_CH=CH_2_), 1.19 ppm (d, *J*=6.6 Hz, 3 H, H-6′′′′); ^13^C NMR (126 MHz, CDCl_3_): *δ*=170.1 (C=O), 170.0 (C=O), 169.9 (C=O), 168.7 (C=O), 139.10 (Ar), 139.06 (*C*H=CH_2_), 138.8 (Ar), 138.75 (Ar), 138.65 (Ar), 138.57 (Ar), 138.56 (Ar), 138.4 (Ar), 138.2 (Ar), 137.6 (Ar), 133.9 (Ar), 131.3 (Ar), 128.7 (Ar), 128.4 (Ar), 128.27 (Ar), 128.25 (2C, Ar), 128.16 (Ar), 128.13 (Ar), 128.11 (Ar), 128.02 (Ar), 128.0 (Ar), 127.85 (Ar), 127.83 (Ar), 127.75 (Ar), 127.5 (Ar), 127.4 (Ar), 127.2 (2C, Ar), 127.1 (Ar), 127.0 (Ar), 126.6 (Ar), 126.3 (Ar), 123.3 (Ar), 114.2 (CH=*C*H_2_), 103.5 (C-1), 102.0 (C-1′), 99.6 (C-1′′′), 99.0 (C-1′′), 97.6 (C-1′′′′), 83.5 (C-3), 82.9 (C-2′), 81.8 (C-2), 79.7 (C-3′′′′), 78.1 (C-3′), 77.2 (C-4′′′′), 75.9 (C-4), 75.4 (O*C*H_2_Ph), 75.3 (C-4′′), 75.1 (C-5′′), 74.9 (O*C*H_2_Ph), 74.75, 74.72 (C-2′′′′, C-5), 74.21, 74.16, 73.8, 73.4, 73.0, 72.9 (6×O*C*H_2_Ph), 72.64, 72.60 (C-3′′, C-5′), 72.4 (O*C*H_2_Ph), 71.0 (C-3′′′), 70.5 (C-5′′′), 69.9 (octenyl OCH_2_), 69.1 (C-2′′′), 68.5, 68.0, 67.9 (C-6, C-6′, C-6′′), 67.5 (C-4′), 66.8 (C-4′′′), 66.6 (C-5′′′′), 60.3 (C-6′′′), 56.2 (C-2′′), 33.7, 29.7, 28.9, 28.8, 26.0 (5×octenyl CH_2_), 20.7, 20.62, 20.56, 20.5 (4×OAc), 16.8 ppm (C-6′′′′); HRMS (ESI): *m*/*z* [*M*+Na]^+^ calcd for C_117_H_131_NNaO_30_: 2052.8648, found: 2052.8612.

**7-Octen-1-yl 2,3,4–6-tetra-*O*-acetyl-β-d-galactopyranosyl-(1→4)-[2,3,4-tri-*O*-benzyl-α-L-fucopyranosyl-(1→3)]-6-*O*-benzyl-2-deoxy-2-phthalimido-β-d-glucopyranosyl-(1→3)-4-*O*-acetyl-2,6-di-*O*-benzyl-β-d-galactopyranosyl-(1→4)-2,3,6-tri-*O*-benzyl-β-d-glucopyranoside (12′)**: A solution of **12** (10 mg, 4.9 μmol) in pyridine (1 mL) and Ac_2_O (0.5 mL, 5.3 mmol) was stirred overnight at RT, concentrated in vacuo, and the residue subjected to flash chromatography (5:2 *v*/*v* hexane/EtOAc) to afford **12’** as a white foam (9.5 mg, 93 %): *R*_f_=0.45 (5:3 *v*/*v* hexane/EtOAc); [*α*]_D_=+4.2 (*c*=0.8, CHCl_3_); ^1^H NMR (600 MHz, CDCl_3_): *δ*=7.45–7.09 (m, 45 H, Ar), 7.02–7.01 (m, 2 H, Ar), 6.87–6.85 (m, 2 H, Ar), 5.80 (ddt, *J*=17.0, 10.3, 6.7 Hz, 1 H, C*H*=CH_2_), 5.46 (dd, *J*=3.6, 0.6 Hz, 1 H, H-4′), 5.28 (dd, *J*=3.6, 1.0 Hz, 1 H, H-4′′′), 5.26 (d, *J*=8.2 Hz, 1 H, H-1′′), 5.04 (dd, *J*=10.4, 8.2 Hz, 1 H, H-2′′′), 5.00–4.97 (m, 1 H, CH=C*H_2_*), 4.95–4.92 (m, 2 H, CH=C*H_2_*, OC*H*_2_Ph), 4.90 (A of ABq, *J*=10.5 Hz, 1 H, OC*H*_2_Ph), 4.86–4.83 (m, 3 H, H-1′′′, H-3′′′, OC*H*_2_Ph), 4.81, 4.57 (ABq, *J*=11.8 Hz, 2 H, 2×OC*H*_2_Ph), 4.78 (d, *J*=3.6 Hz, 1 H, H-1′′′′), 4.75 (dd, *J*=10.2, 9.0 Hz, 1 H, H-3′′), 4.69–4.63 (m, 5 H, H-5′′′′, 4×OC*H*_2_Ph), 4.55 (A of ABq, *J*=11.7 Hz, 1 H, OC*H*_2_Ph), 4.47 (A of ABq, *J*=12.2 Hz, 1 H, OC*H*_2_Ph), 4.43–4.40 (m, 3 H, H-2′′, 2 × OC*H*_2_Ph), 4.30–4.26 (m, 3 H, H-1′, 2 × OC*H*_2_Ph), 4.23–4.15 (m, 5 H, H-1, H-4′′, H-6a′′′, 2×OC*H*_2_Ph), 4.00 (A of ABq, *J*=11.8 Hz, 1 H, OC*H*_2_Ph), 3.97 (dd, *J*=10.8, 5.7 Hz, 1 H, H-6b′′′), 3.93 (dd, *J*=5.4, 3.0 Hz, 1 H, H-6a′′), 3.89–3.83 (m, 4 H, H-3′′′′, H-4, H-6b′′, octenyl OCH_2_), 3.77 (dd, *J*=10.2, 3.7 Hz, 1 H, H-2′′′′), 3.63–3.60 (m, 2 H, H-4′′′′, H-5′′′), 3.56–3.52 (m, 2 H, H-3′, H-5′′), 3.47–3.41 (m, 3 H, H-5′, H-6a, octenyl OCH_2_), 3.38 (m, 2 H, H-3, H-6a′), 3.33–3.29 (m, 4 H, H-2, H-2′, H-6b, H-6b′), 2.98–2.96 (ddd, *J*=9.6, 3.6, 1.8 Hz, 1 H, H-5), 2.09 (s, 3 H, OAc), 2.05 (s, 3 H, OAc), 2.03–2.02 (m, 5 H, OAc, OCH_2_CH_2_(CH_2_)_3_C*H_2_*CH=CH_2_), 1.97 (s, 3 H, OAc), 1.85 (s, 3 H, OAc), 1.63–1.59 (m, 2 H, OCH_2_C*H_2_*(CH_2_)_3_CH_2_CH=CH_2_), 1.39–1.29 (m, 6 H, OCH_2_CH_2_(C*H_2_*)_3_CH_2_CH=CH_2_), 1.21 ppm (d, *J*=6.0 Hz, 3 H, C-6′′′′); ^13^C NMR (126 MHz; CDCl_3_): *δ*=170.02 (C=O), 170.01 (C=O), 169.96 (C=O), 169.86 (C=O), 168.8 (C=O), 139.12 (Ar), 139.05 (*C*H=CH_2_), 138.89 (Ar), 138.69 (Ar), 138.68 (Ar), 138.4 (Ar), 138.28 (Ar), 138.27 (Ar), 138.23 (Ar), 138.19 (Ar), 133.8 (Ar), 131.3 (Ar), 128.5 (Ar), 128.31 (Ar), 128.26 (Ar), 128.15 (Ar), 128.13 (Ar), 128.09 (Ar), 128.01 (Ar), 127.94 (Ar), 127.92 (Ar), 127.90 (Ar), 127.83 (Ar), 127.79 (2C, Ar), 127.78 (Ar), 127.53 (Ar), 127.48 (Ar), 127.41 (Ar), 127.3 (Ar), 127.13 (Ar), 127.11 (Ar), 127.00 (Ar), 126.9 (Ar), 126.4 (Ar), 123.3 (Ar), 114.2 (CH=*C*H_2_), 103.6 (C-1), 102.0 (C-1′), 99.5 (C-1′′′), 99.1 (C-1′′), 97.2 (C-1′′′′), 82.6 (C-3), 81.6, 78.84, 78.79 (C-2, C-2′, C-3′), 79.7 (C-3′′′′), 77.2 (C-4′′′′), 75.7 (C-4), 75.4 (C-5′′), 75.2 (O*C*H_2_Ph), 74.96 (C-4′′), 74.92 (O*C*H_2_Ph), 74.7, 74.5 (C-5, C-2′′′′), 74.20, 74.15, 73.6, 73.50, 73.0, 72.7 (6×O*C*H_2_Ph), 72.6 (C-5′), 72.4 (O*C*H_2_Ph), 72.0 (C-3′′), 71.0 (C-3′′′), 70.4 (C-5′′′), 69.94 (C-4′), 69.90 (octenyl OCH_2_), 69.0 (C-2′′′), 68.3, 67.7, 67.6 (C-6, C-6′, C-6′′), 66.8 (C-4′′′), 66.4 (C-5′′′′), 60.2 (C-6′′′), 56.6 (C-2′′), 33.7, 29.7, 28.9, 28.8, 26.0 (5×octenyl CH_2_), 20.83, 20.75, 20.64, 20.56, 20.54 (5×OAc), 16.7 ppm (C-6′′′′); HRMS (ESI): *m*/*z* [*M*+Na]^+^ calcd for C_119_H_133_NNaO_31_: 2094.8754, found: 2094.8751.

**7-Octen-1-yl β-d-galactopyranosyl-(1→4)-[2,3,4-tri-*O*-benzyl-α-L-fucopyranosyl-(1→3)]-6-*O*-benzyl-2-deoxy-2-acetamido-β-d-glucopyranosyl-(1→3)-2,6-di-*O*-benzyl-β-d-galactopyranosyl-(1→4)-2,3,6-tri-*O*-benzyl-β-d-glucopyranoside (13)**: A solution of pentasaccharide **12** (352 mg, 0.17 mmol) in *n*-butanol (15 mL) was treated with ethylenediamine (3 mL, 44.9 mmol), followed by stirring at 100 °C for 20 h. The solution was concentrated in vacuo to dryness. The crude residue was dissolved in CH_2_Cl_2_/MeOH (1:2 *v*/*v*, 6 mL), to which Ac_2_O (1 mL) and Et_3_N (1 mL) were added. After stirring at RT for 5 h, the solution was concentrated, and the residue subjected to flash chromatography (3:2 *v*/*v* toluene/acetone). Further purification by C_18_ chromatography (1:1 *v*/*v* MeOH/H_2_O→MeOH) afforded **13** as a white foam (255 mg, 83 %). *R*_f_=0.51 (4:5 *v*/*v* toluene/acetone); [*α*]_D_=−19.6 (*c*=1.0, CHCl_3_); ^1^H NMR (600 MHz, CDCl_3_): *δ*=7.42–7.19 (m, 45 H), 5.80 (ddt, *J*=17.0, 10.3, 6.7 Hz, 1 H, C*H*=CH_2_), 5.67 (d, *J*=7.2 H, 1 H, NH), 5.16 (d, *J*=7.5 Hz, 1 H, H-1′′), 5.05 (d, *J*=3.6 Hz, 1 H, H-1′′′′), 5.00–4.97 (m, 2 H, CH=C*H_2_*, OC*H*_2_Ph), 4.94–4.87 (m, 4 H, CH=C*H_2_*, 3×OC*H*_2_Ph), 4.74–4.70 (m, 4 H, 4×OC*H*_2_Ph), 4.66–4.63 (m, 2 H, 2×OC*H*_2_Ph), 4.60–4.55 (m, 3 H, 3×OC*H*_2_Ph), 4.53, 4.47 (ABq, *J*=12.2 Hz, 2 H, 2×OC*H*_2_Ph), 4.44–4.38 (m, 4 H, H-1′, H-1′′′, 2×OC*H*_2_Ph), 4.35–4.32 (m, 2 H, H-1, H-3′′), 4.29 (A of ABq, *J*=12.0 Hz, 1 H, OC*H*_2_Ph), 4.12–4.08 (m, 2 H, H-5′′′′, OH), 4.05–4.01 (m, 3 H, H-2′′′′), 3.94–3.86 (m, 5 H, H-3′′′′, H-6a′′′, octenyl OCH_2_), 3.78–3.76 (m, 1 H, H-4′′), 3.74–3.65 (m, 6 H, H-6b′′′), 3.62–3.58 (m, 2 H, H-4′′′′), 3.54–3.46 (m, 7 H, H-2′, H-2′′′, H-3, H-3′, octenyl OCH_2_), 3.44–3.33 (m, 4 H, H-2, H-2′′), 3.30–3.27 (m, 1 H), 2.99–2.97 (m, 2 H, OH), 2.72 (br s, 1 H, OH), 2.05–2.01 (m, 2 H, OCH_2_CH_2_(CH_2_)_3_C*H_2_*CH=CH_2_), 1.64–1.62 (m, 2 H, OCH_2_C*H_2_*(CH_2_)_3_CH_2_CH=CH_2_), 1.40–1.32 (m, 9 H, NHAc, OCH_2_CH_2_(C*H_2_*)_3_CH_2_CH=CH_2_), 1.11 ppm (d, *J*=6.0 Hz, 3 H, H-6′′′′); ^13^C NMR (126 MHz, CDCl_3_): *δ*=170.9 (C=O), 139.2 (Ar), 139.1 (*C*H=CH_2_), 139.0 (Ar), 138.7 (Ar), 138.44 (Ar), 138.43 (Ar), 138.38 (Ar), 138.2 (Ar), 137.5 (Ar), 128.6 (Ar), 128.51 (Ar), 128.47 (Ar), 128.31 (Ar), 128.27 (Ar), 128.25 (Ar), 128.20 (Ar), 128.1 (Ar), 128.04 (2C, Ar), 128.01 (Ar), 127.91 (Ar), 127.86 (Ar), 127.71 (Ar), 127.67 (Ar), 127.66 (Ar), 127.63 (Ar), 127.53 (Ar), 127.51 (Ar), 127.46 (Ar), 127.39 (Ar), 127.37 (Ar), 127.2 (Ar), 114.2 (CH=*C*H_2_), 103.6 (C-1), 102.2 (C-1′), 100.1 (C-1′′′), 99.7 (C-1′′), 98.0 (C-1′′′′), 82.9 (C-3), 82.3 (C-2′), 81.9 (C-2), 79.4, 79.0 (C-3′, C-3′′′′), 77.2 (C-4′′′′), 76.6, 76.5, 76.4, 76.0, 75.3 (O*C*H_2_Ph), 75.14, 75.08, 74.95 (O*C*H_2_Ph), 74.93 (O*C*H_2_Ph), 74.8, 74.7 (O*C*H_2_Ph), 74.1 (O*C*H_2_Ph), 73.7, 73.6, 73.4, 73.2 (3×O*C*H_2_Ph), 72.9, 72.4 (O*C*H_2_Ph), 71.7, 70.0, 69.9, 68.9, 68.4 (C-6, C-6′, C-6′′, octenyl OCH_2_), 69.3, 67.9, 67.5 (C-5′′′′), 63.0 (C-6′′′), 57.5 (C-2′′), 33.7, 29.7, 28.95, 28.86, 26.0 (5×octenyl CH_2_), 22.9 (NHAc), 16.7 ppm (C-6′′′′); HRMS (ESI): *m*/*z* [*M*+Na]^+^ calcd for C_103_H_123_NNaO_25_: 1796.8276, found: 1796.8254.

**7-Octen-1-yl β-d-galactopyranosyl-(1→4)-[α-L-fucopyranosyl-(1→3)]-2-acetamido-2-deoxy-β-d-glucopyranosyl-(1→3)-β-d-galactopyranosyl-(1→4)-β-d-glucopyranoside (14)**: Sodium was added to freshly collected liquid ammonia (∼8 mL) at −78 °C until the blue colour of the solution persisted. A solution of **13** (144 mg, 0.73 mmol) in THF (4 mL) and MeOH (30 μL) was added drop wise at −78 °C. After 2 h, MeOH (5 mL) was added and the solution was concentrated in vacuo to dryness. The residue was dissolved in MeOH (30 mL), neutralized with Amberlite IR120 H^**+**^ ion-exchange resin, filtered and concentrated. The crude residue was purified by C_18_ chromatography using gradient elution (H_2_O→3:7 *v*/*v* MeOH/H_2_O) to give **14** as a white solid (76 mg, 97 %): [*α*]_D_=−45.6 (*c*=0.9, MeOH); ^1^H NMR (600 MHz, D_2_O): *δ*=5.91 (ddt, *J*=17.2, 10.4, 6.7 Hz, 1 H, C*H*=CH_2_), 5.11 (d, *J*=4.0 Hz, 1 H, H-1′′′′), 5.06–5.02 (m, 1 H, CH=C*H_2_*), 4.97–4.95 (m, 1 H, CH=C*H_2_*), 4.82 (q, *J*=6.7 Hz, 1 H, H-5′′′′), 4.70 (d, *J*=8.3 Hz, 1 H, H-1′′), 4.46 (d, *J*=7.8 Hz, 1 H, H-1′′′), 4.45 (d, *J*=7.8 Hz, 1 H, H-1′), 4.42 (d, *J*=7.8 Hz, 1 H, H-1), 4.14 (d, *J*=3.5 Hz, 1 H), 3.96–3.84 (m, 9 H), 3.79–3.55 (m, 17 H), 3.48 (dd, *J*=9.8, 7.8 Hz, H-2′), 3.28 (dd, *J*=9.6, 7.8 Hz, 1 H, H-2′′′), 2.07–2.03 (m, 2 H, OCH_2_CH_2_(CH_2_)_3_C*H_2_*CH=CH_2_), 2.01 (s, 3 H, NHAc), 1.63–1.59 (m, 2 H, OCH_2_C*H_2_*(CH_2_)_3_CH_2_CH=CH_2_), 1.41–1.30 (m, 6 H, OCH_2_CH_2_(C*H_2_*)_3_CH_2_CH=CH_2_), 1.16 ppm (d, *J*=6.6 Hz, 3 H, H-6′′′′); ^13^C NMR (126 MHz, D_2_O): *δ*=175.7 (C=O), 141.3 (*C*H=CH_2_), 115.0 (CH=*C*H_2_), 103.9 (C-1), 103.5, 103.0, 102.8 (C-1′, C-1′′, C-1′′′), 99.6 (C-1′′′′), 83.1, 79.4, 76.1, 75.9, 75.8, 75.7 (2C), 75.4, 74.1, 73.8, 73.5, 72.9, 72.0, 71.7, 70.9, 70.2, 69.33, 69.28, 68.7, 67.7 (C-5′′′′), 62.5, 61.9, 61.1, 60.6 (C-6, C-6′, C-6′′, C-6′′′), 57.0 (C-2′′), 34.0 (octenyl CH_2_), 29.6 (octenyl CH_2_), 29.0 (2C, octenyl CH_2_), 25.8 (octenyl CH_2_), 23.2 (NHAc), 16.3 ppm (C-6′′′′); HRMS (ESI): *m*/*z* [*M*+Na]^+^ calcd for C_40_H_69_NNaO_25_: 986.4051, found: 986.4047.

**8-[(2-Aminoethyl)thiol]-1-octyl β-d-galactopyranosyl-(1→4)-[α-L-fucopyranosyl-(1→3)]-2-acetamido-2-deoxy-β-d-glucopyranosyl-(1→3)-β-d-galactopyranosyl-(1→4)-β-d-glucopyranoside (15)**: Compound **14** (38 mg, 0.039 mmol) and cysteamine hydrochloride (44 mg, 0.39 mmol) were dissolved in dry MeOH (0.5 mL) in a quartz tube. The solution was degassed and the tube was filled with argon. After irradiation with UV light for 2.5 h, the solution was concentrated and subjected to C_18_ chromatography using gradient elution (0.5 % aq AcOH→3:7 *v*/*v* MeOH/0.5 % aq AcOH) to afford the corresponding amine salt **15** (42 mg, quantitative). [*α*]_D_=−41.1 (*c*=0.9, MeOH); ^1^H NMR (600 MHz, D_2_O): *δ*=5.11 (d, *J*=3.9 Hz, 1 H, H-1′′′′), 4.83–4.80 (m, 1 H, H-5′′′′), 4.70 (d, *J*=8.4 Hz, 1 H, H-1′′), 4.46 (d, *J*=7.8 Hz, 1 H, H-1′′′), 4.45 (d, *J*=7.8 Hz, 1 H, H-1′), 4.42 (d, *J*=7.8 Hz, 1 H, H-1), 4.14 (d, *J*=2.9 Hz, 1 H), 3.96–3.84 (m, 9 H), 3.79–3.55 (m, 17 H), 3.48 (dd, *J*=9.6, 7.8 Hz, 1 H, H-2′), 3.29–3.26 (m, 1 H, H-2′′′), 3.20 (t, *J*=6.7 Hz, 2 H, SCH_2_C*H_2_*N), 2.83 (t, *J*=6.7 Hz, 2 H, SC*H_2_*CH_2_N), 2.58 (t, *J*=7.3 Hz, 2 H, SC*H_2_*(CH_2_)_6_CH_2_O), 2.01 (s, 3 H, NHAc), 1.63–1.56 (m, 4 H, OCH_2_C*H_2_*(CH_2_)_4_C*H_2_*CH_2_S), 1.38–1.31 (m, 8 H, OCH_2_CH_*2*_(C*H_2_*)_4_CH_2_CH_2_S), 1.16 ppm (d, *J*=6.6 Hz, 3 H, H-6′′′′); ^13^C NMR (126 MHz, D_2_O): *δ*=175.7 (C=O), 103.9 (C-1), 103.5, 103.0, 102.8 (C-1′, C-1′′, C-1′′′), 99.6 (C-1′′′′), 83.1, 79.4, 76.1, 75.90, 75.86, 75.7 (2C), 75.5, 74.1, 73.8, 73.5, 72.9, 72.0, 71.7, 70.9, 70.2, 69.34, 69.27, 68.7, 67.7 (C-5′′′′), 62.5, 61.9, 61.1, 60.6 (C-6, C-6′, C-6′′, C-6′′′), 57.0 (C-2′′), 39.4 (SCH_2_*C*H_2_N), 31.7 (S*C*H_2_(CH_2_)_6_CH_2_O), 29.7 (SCH_2_(*C*H_2_)_6_CH_2_O), 29.5 (S*C*H_2_CH_2_N), 29.3, 29.1(2C), 28.8, 25.9 (5×SCH_2_(*C*H_2_)_6_CH_2_O), 23.2 (NHAc), 16.3 ppm (C-6′′′′); HRMS (ESI): *m*/*z* [*M*+H]^+^ calcd for C_42_H_77_N_2_O_25_S: 1041.4531, found: 1041.4517.

**Preparation of HSA conjugate**: Compound **1** (1.5 mg) was dissolved in DMF (15 μL) and injected into a solution of human serum albumin (HSA; 1.5 mg) in phosphate buffer (0.3 mL, pH 7.5). The reaction was left at RT for one day, and the mixture was dialyzed against deionized H_2_O (5×4 L). A white solid was obtained after lyophilization. The degree of incorporation of the pentasaccharide into the glycoconjugate was determined to be 21 by MALDI-TOF MS.

## Supplementary material

As a service to our authors and readers, this journal provides supporting information supplied by the authors. Such materials are peer reviewed and may be re-organized for online delivery, but are not copy-edited or typeset. Technical support issues arising from supporting information (other than missing files) should be addressed to the authors.

## References

[1] Ko AI, Dräger UC, Harn DA (1990). Proc. Natl. Acad. Sci. USA.

[2] Stahl B, Thurl S, Zeng JR, Karas M, Hillenkamp F, Steup M, Sawatzki G (1994). Anal. Biochem.

[3] Dutta P, Hullett DA, Roenneburg DA, Torrealba JR, Sollinger HW, Harn DA, Burlingham WJ (2010). Transplantation.

[4] Freeman GJ, Long AJ, Iwai Y, Bourque K, Chernova T, Nishimura H, Fitz LJ, Malenkovich N, Okazaki T, Byrne MC, Horton HF, Fouser L, Carter L, Ling V, Bowman MR, Carreno BM, Collins M, Wood CR, Honjo T (2000). J. Exp. Med.

[5] Fan X, Ang A, Pollock-BarZiv SM, Dipchand AI, Ruiz P, Wilson G, Platt JL, West LJ (2004). Nat. Med.

[6] Slaney AM, Wright VA, Meloncelli PJ, Harris KD, West LJ, Lowary TL, Buriak JM (2011). ACS Appl. Mater. Interfaces.

[7] Zhang Y-M, Esnault J, Mallet J-M, Sinaÿ P (1999). J. Carbohydr. Chem.

[8] Zhang Y, Dong D, Qu H, Sollogoub M, Zhang Y (2011). Eur. J. Org. Chem.

[9] Chao C-S, Li C-W, Chen M-C, Chang S-S, Mong K-KT (2009). Chem. Eur. J.

[10] Zhang Z, Ollmann IR, Ye X-S, Wischnat R, Baasov T, Wong C-H (1999). J. Am. Chem. Soc.

[11] Johnsson R, Olsson D, Ellervik U (2008). J. Org. Chem.

[12] Cheng H, Cao X, Xian M, Fang L, Cai TB, Ji JJ, Tunac JB, Sun D, Wang PG (2005). J. Med. Chem.

[13] Ellervik U, Magnusson G (1998). J. Org. Chem.

[14] Guillemineau M, Auzanneau F-I (2012). Carbohydr. Res.

[15] Bock K, Pedersen C (1974). J. Chem. Soc. Perkin Trans. 2.

[16] Codée JDC, Litjens REJN, den Heeten R, Overkleeft HS, van Boom JH, van der Marel GA (2003). Org. Lett.

[17] Dondoni A, Massi A, Nanni P, Roda A (2009). Chem. Eur. J.

[18] Wu X, Ling C-C, Bundle DR (2004). Org. Lett.

