# Supplementary material for: Synthesis of the Tolerance-Inducing Oligosaccharide Lacto-N-Fucopentaose III Bearing an Activated Linker
Source: ChemistryOpen. 2013 Jul 11;2(4):156–63. doi: 10.1002/open.201300024 (PMC3775522; doi:10.1002/open.201300024)
Supplement: Supplementary file 1 [file open0002-0156-SD1.pdf]

ChemistryOPEN

Supporting Information

© 2013 The Authors. Published by Wiley-VCH Verlag GmbH & Co. KGaA, Weinheim

Synthesis of the Tolerance-Inducing Oligosaccharide Lacto-*N*-Fucopentaose III Bearing an Activated Linker

Junfeng Zhang, Lu Zou, and Todd L. Lowary*[a]

open_201300024_sm_miscellaneous_information.pdf

Table of contents

^{1}H NMR and ^{13}C NMR spectra of **1** S2–S3
^{1}H NMR and ^{13}C NMR spectra of **2** S4–S5
^{1}H NMR and ^{13}C NMR spectra of **3** S6–S7
^{1}H NMR and ^{13}C NMR spectra of **6** S8–S9
^{1}H NMR and ^{13}C NMR spectra of **7** S10–S11
^{1}H NMR, ^{13}C NMR and HMBC spectra of **10** S12–S14
^{1}H NMR and ^{13}C NMR spectra of **12** S15–S16
^{1}H NMR and ^{13}C NMR spectra of **12′** S17–S18
^{1}H NMR and ^{13}C NMR spectra of **13** S19–S20
^{1}H NMR and ^{13}C NMR spectra of **14** S21–S22
^{1}H NMR and ^{13}C NMR spectra of **15** S23–S24
MALDI mass spectrum of HSA conjugate S25

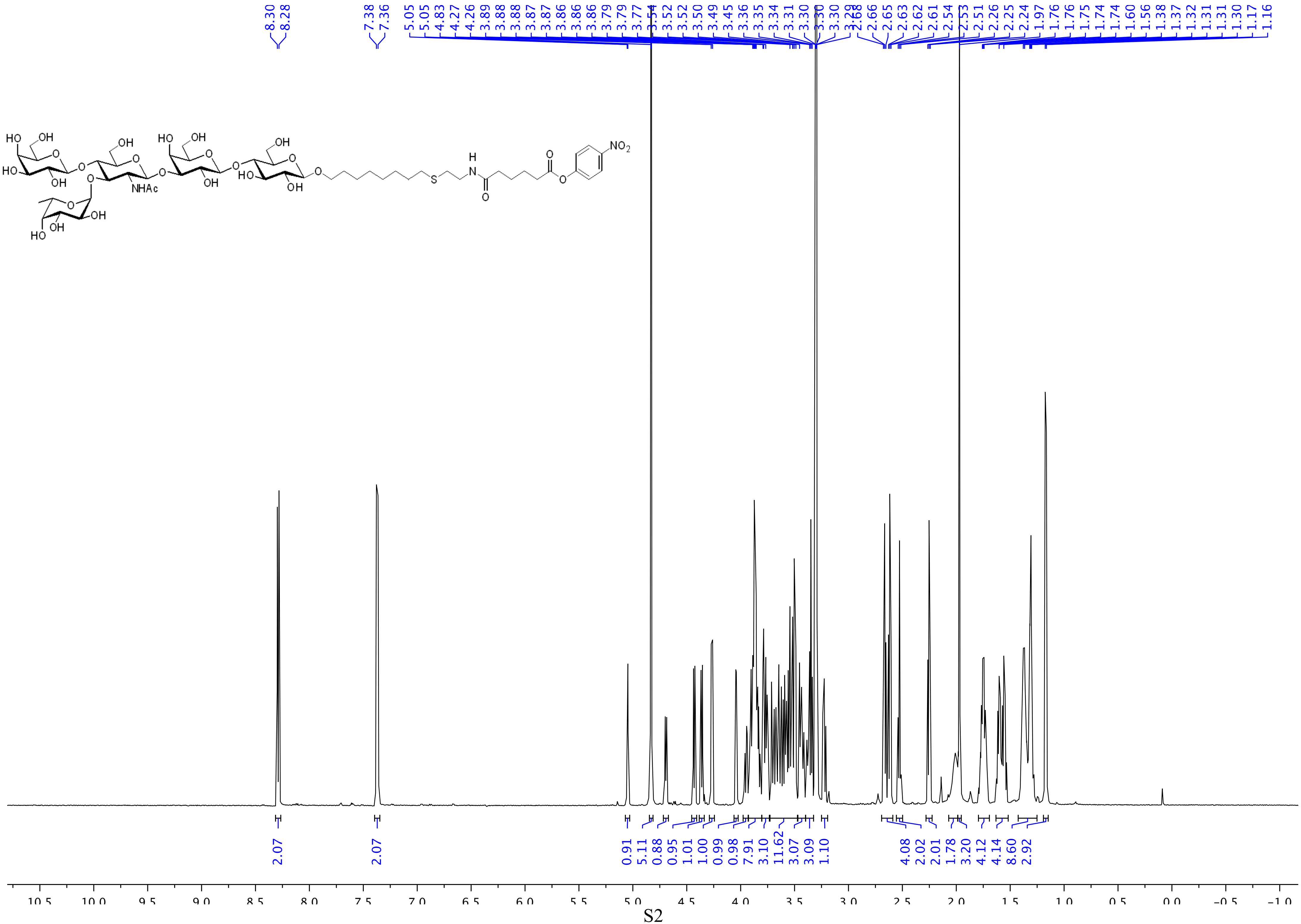

8.30
8.28
7.38
7.36
5.05
5.05
4.83
4.27
4.26
3.89
3.88
3.88
3.87
3.87
3.86
3.86
3.79
3.77
3.54
3.52
3.52
3.50
3.49
3.45
3.36
3.35
3.34
3.31
3.30
3.30
3.30
3.29
2.68
2.66
2.65
2.63
2.62
2.61
2.54
2.53
2.51
2.26
2.25
2.24
1.97
1.76
1.76
1.75
1.74
1.74
1.60
1.56
1.38
1.37
1.32
1.31
1.31
1.30
1.17
1.16
HO
OH
NHAc
NO2
S
H
N
O
2.07
2.07
0.91
5.11
0.88
0.95
1.01
1.00
0.99
0.98
7.91
3.10
11.62
3.07
3.09
1.10
4.08
2.02
2.01
1.78
3.20
4.12
4.14
8.60
2.92
10.5
10.0
9.5
9.0
8.5
8.0
7.5
7.0
6.5
6.0
5.5
5.0
4.5
4.0
3.5
3.0
2.5
2.0
1.5
1.0
0.5
0.0
-0.5
-1.0

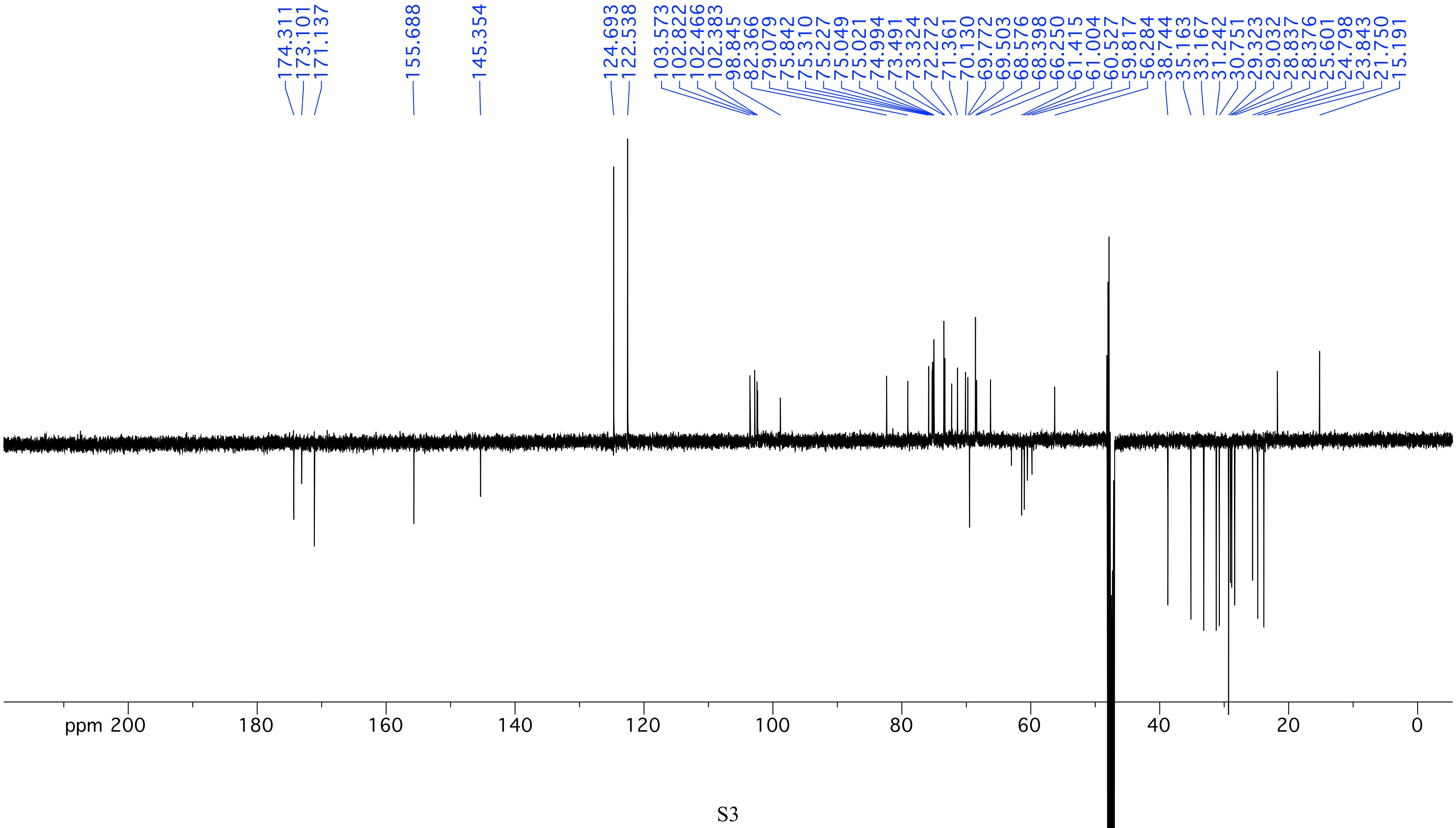

174.311
173.101
171.137
155.688
145.354
124.693
122.538
103.573
102.822
102.466
102.383
98.845
82.366
79.079
75.842
75.310
75.227
75.049
75.021
74.994
73.491
73.324
72.272
71.361
70.130
69.772
69.503
68.576
68.398
66.250
61.415
61.004
60.527
59.817
56.284
38.744
35.163
33.167
31.242
30.751
29.323
29.032
28.837
28.376
25.601
24.798
23.843
21.750
15.191
ppm 200
180
160
140
120
100
80
60
40
20
0

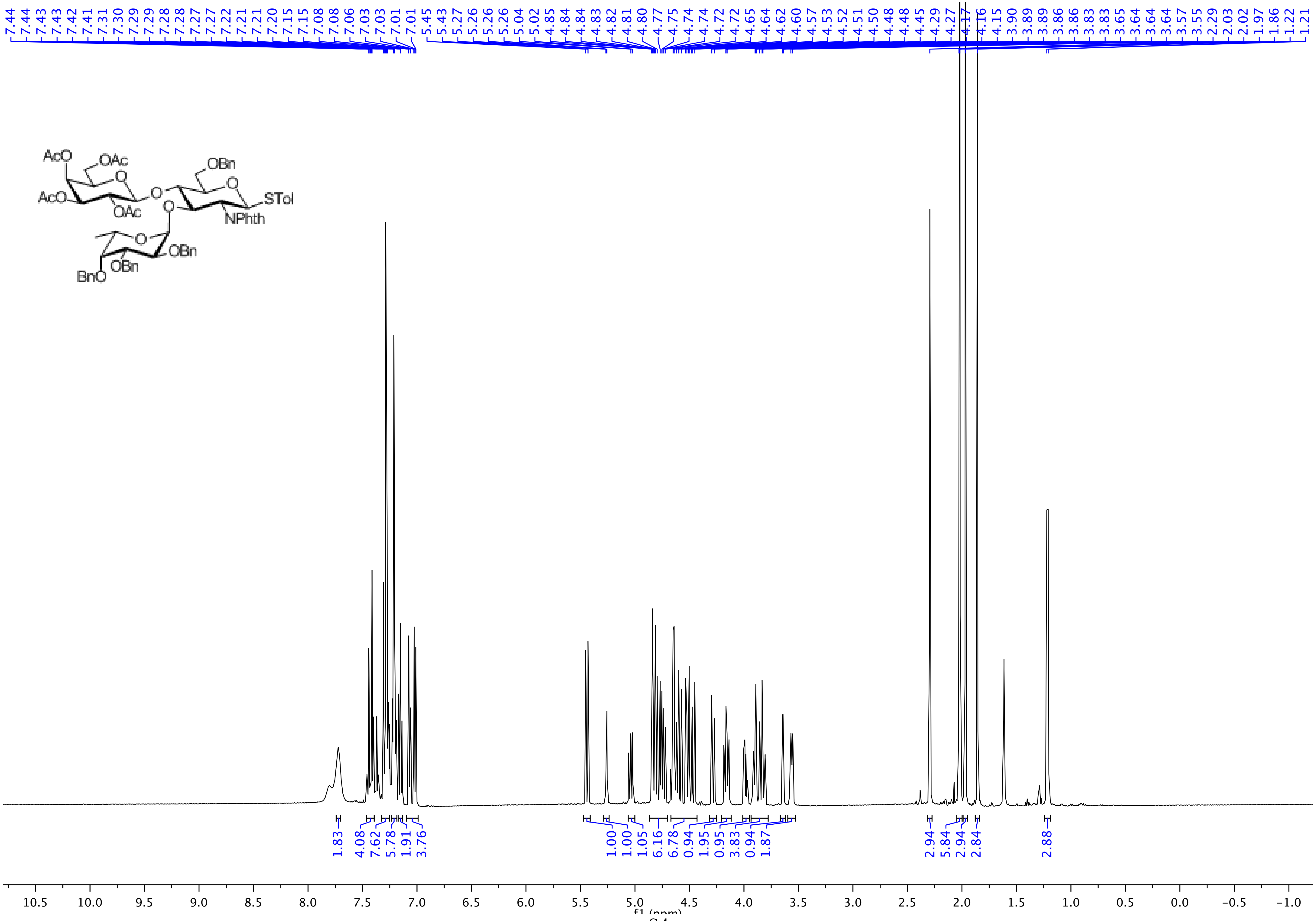
7.44 7.44 7.43 7.43 7.42 7.41 7.31 7.30 7.29 7.29 7.28 7.28 7.27 7.27 7.22 7.21 7.21 7.20 7.15 7.15 7.08 7.08 7.06 7.03 7.03 7.01 7.01 5.45 5.43 5.27 5.26 5.26 5.26 5.04 5.02 4.85 4.84 4.84 4.83 4.82 4.81 4.80 4.77 4.75 4.74 4.74 4.72 4.72 4.65 4.64 4.62 4.60 4.57 4.53 4.52 4.51 4.50 4.48 4.48 4.45 4.29 4.27 4.17 4.16 4.15 3.90 3.89 3.89 3.86 3.86 3.83 3.83 3.65 3.64 3.64 3.57 3.55 2.29 2.03 2.02 1.97 1.86 1.22 1.21
AcO
OAc
O
OBn
O
STol
AcO
OAc
NPhth
O
O
OBn
OBn
BnO
1.83 4.08 7.62 5.78 1.91 3.76
1.00 1.00 1.05 6.16 6.78 0.94 1.95 0.95 3.83 0.94 1.87
2.94 5.84 2.94 2.84
2.88
10.5 10.0 9.5 9.0 8.5 8.0 7.5 7.0 6.5 6.0 5.5 5.0 4.5 4.0 3.5 3.0 2.5 2.0 1.5 1.0 0.5 0.0 -0.5 -1.0
f1 (ppm)

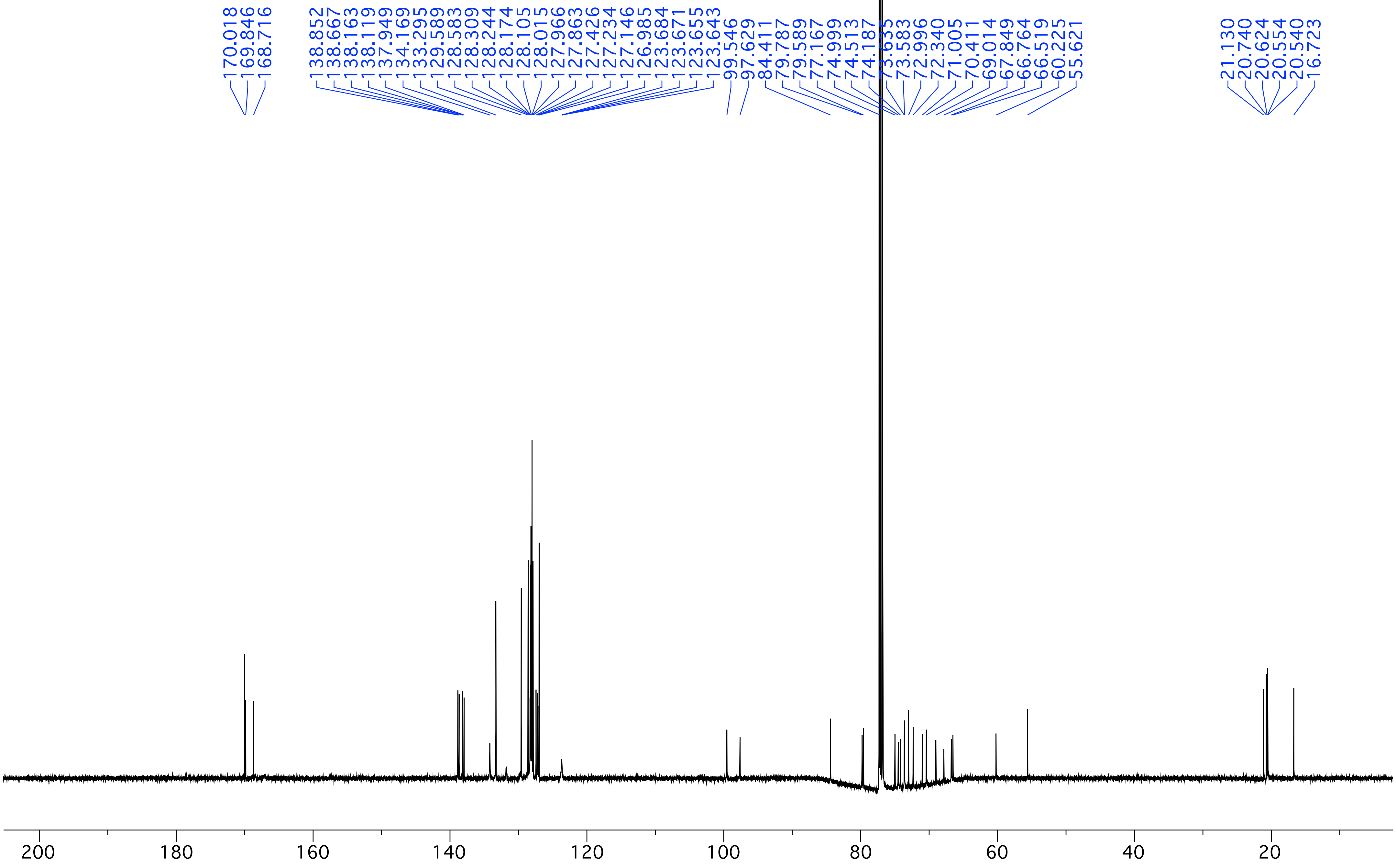
170.018
169.846
168.716
138.852
138.667
138.163
138.119
137.949
134.169
133.295
129.589
128.583
128.309
128.244
128.174
128.105
128.015
127.966
127.863
127.426
127.234
127.146
126.985
123.684
123.671
123.655
123.643
99.546
97.629
84.411
79.787
79.589
77.167
74.999
74.513
74.187
73.635
73.583
72.996
72.340
71.005
70.411
69.014
67.849
66.764
66.519
60.225
55.621
21.130
20.740
20.624
20.554
20.540
16.723
200
180
160
140
120
100
80
60
40
20

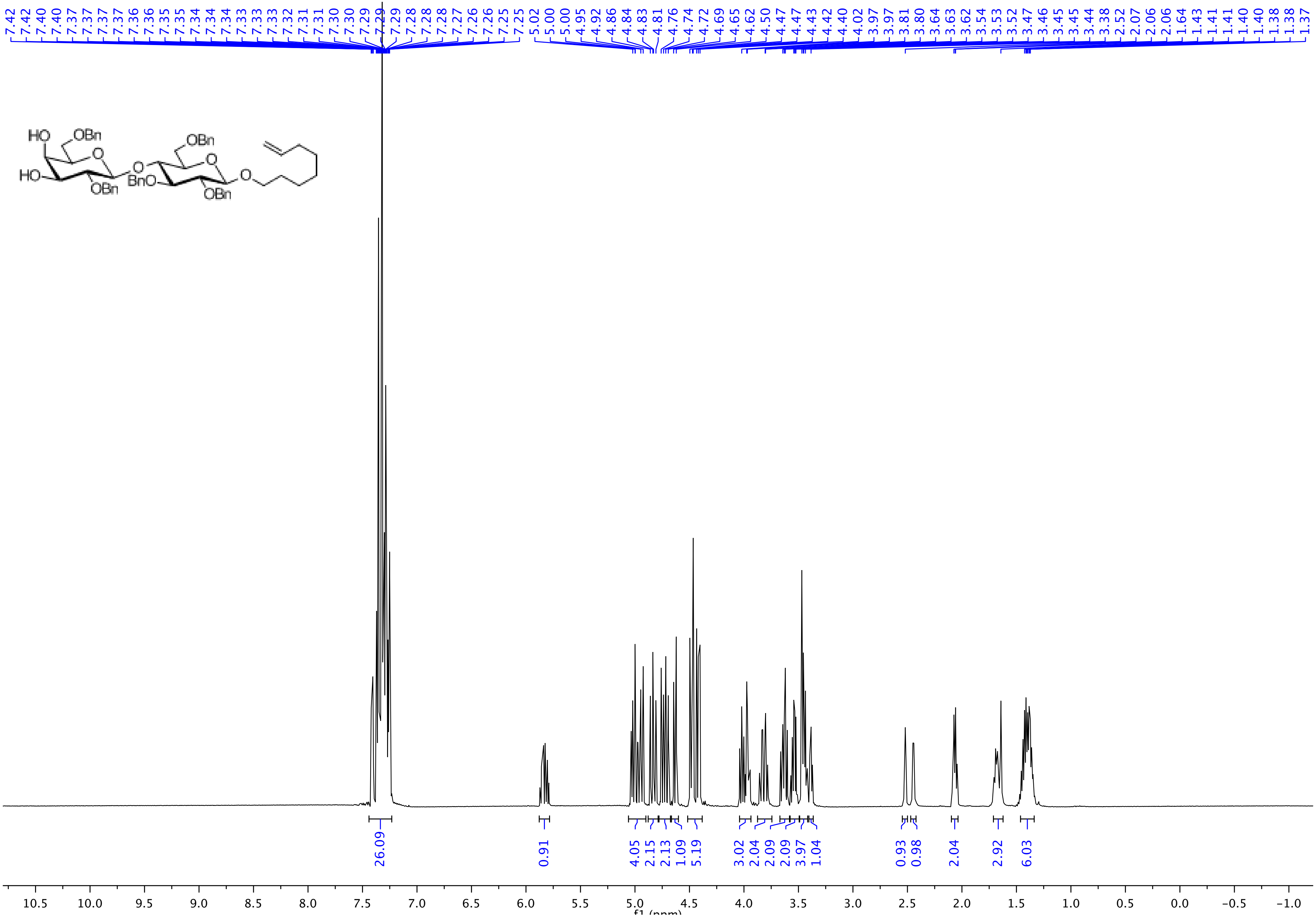
7.42 7.40 7.40 7.37 7.37 7.37 7.37 7.36 7.36 7.35 7.35 7.34 7.34 7.34 7.33 7.33 7.33 7.32 7.31 7.31 7.30 7.30 7.29 7.29 7.29 7.28 7.28 7.28 7.27 7.26 7.26 7.25 7.25 5.02 5.00 5.00 4.95 4.92 4.86 4.84 4.83 4.81 4.76 4.74 4.72 4.69 4.65 4.62 4.50 4.47 4.47 4.43 4.42 4.40 4.02 3.97 3.97 3.81 3.80 3.64 3.63 3.62 3.54 3.53 3.52 3.47 3.46 3.45 3.45 3.44 3.38 2.52 2.07 2.06 2.06 1.64 1.43 1.41 1.41 1.40 1.40 1.38 1.38 1.37
HO
OBn
HO
OBn
BnO
OBn
OBn
O
26.09
0.91
4.05 2.15 2.13 1.09 5.19
3.02 2.04 2.09 2.09 3.97 1.04
0.93 0.98
2.04
2.92
6.03
10.5 10.0 9.5 9.0 8.5 8.0 7.5 7.0 6.5 6.0 5.5 5.0 4.5 4.0 3.5 3.0 2.5 2.0 1.5 1.0 0.5 0.0 −0.5 −1.0
f1 (ppm)

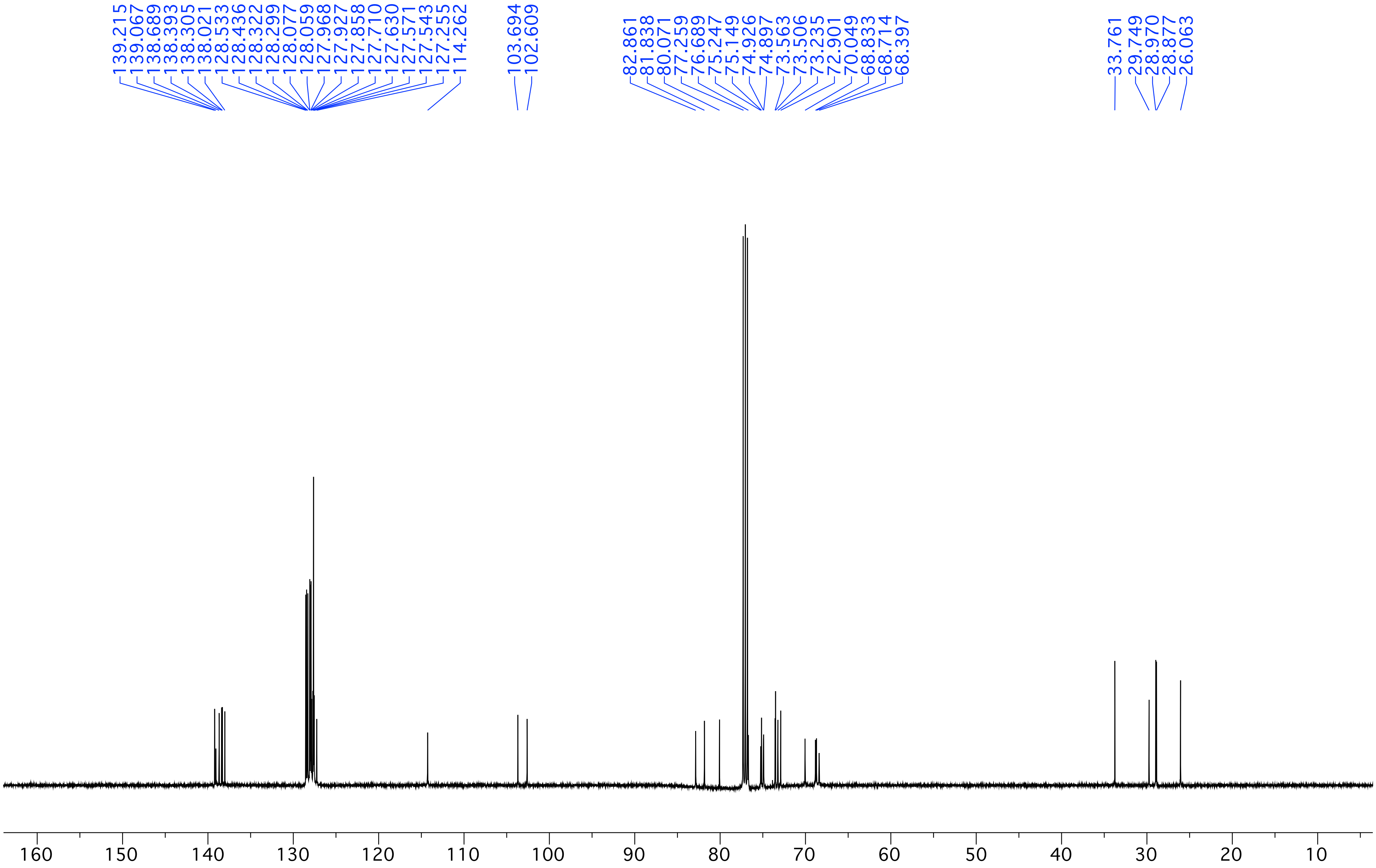
139.215
139.067
138.689
138.393
138.305
138.021
128.533
128.436
128.322
128.299
128.077
128.059
127.968
127.927
127.858
127.710
127.630
127.571
127.543
127.255
114.262
103.694
102.609
82.861
81.838
80.071
77.259
76.689
75.247
75.149
74.926
74.897
73.563
73.506
73.235
72.901
70.049
68.833
68.714
68.397
33.761
29.749
28.970
28.877
26.063
160
150
140
130
120
110
100
90
80
70
60
50
40
30
20
10

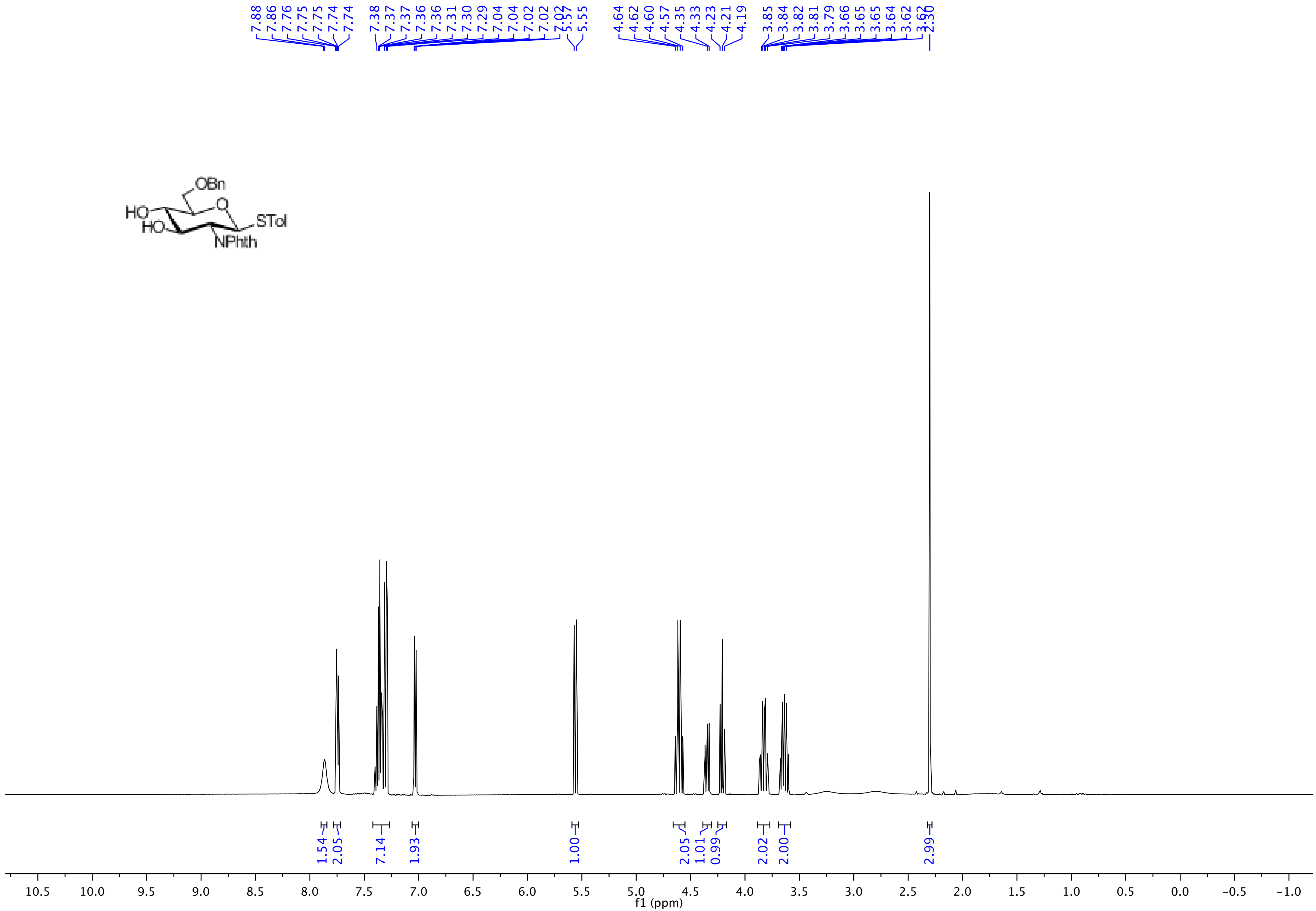
7.88 7.86 7.76 7.75 7.75 7.74 7.74 7.38 7.37 7.37 7.36 7.36 7.31 7.30 7.29 7.04 7.04 7.02 7.02 7.02 5.57 5.55 4.64 4.62 4.60 4.57 4.35 4.33 4.23 4.21 4.19 3.85 3.84 3.82 3.81 3.79 3.66 3.65 3.65 3.64 3.62 3.62 2.30
OBn
HO
HO
O
STol
NPhth
1.54 2.05 7.14 1.93 1.00 2.05 1.01 0.99 2.02 2.00 2.99
10.5 10.0 9.5 9.0 8.5 8.0 7.5 7.0 6.5 6.0 5.5 5.0 4.5 4.0 3.5 3.0 2.5 2.0 1.5 1.0 0.5 0.0 −0.5 −1.0
f1 (ppm)

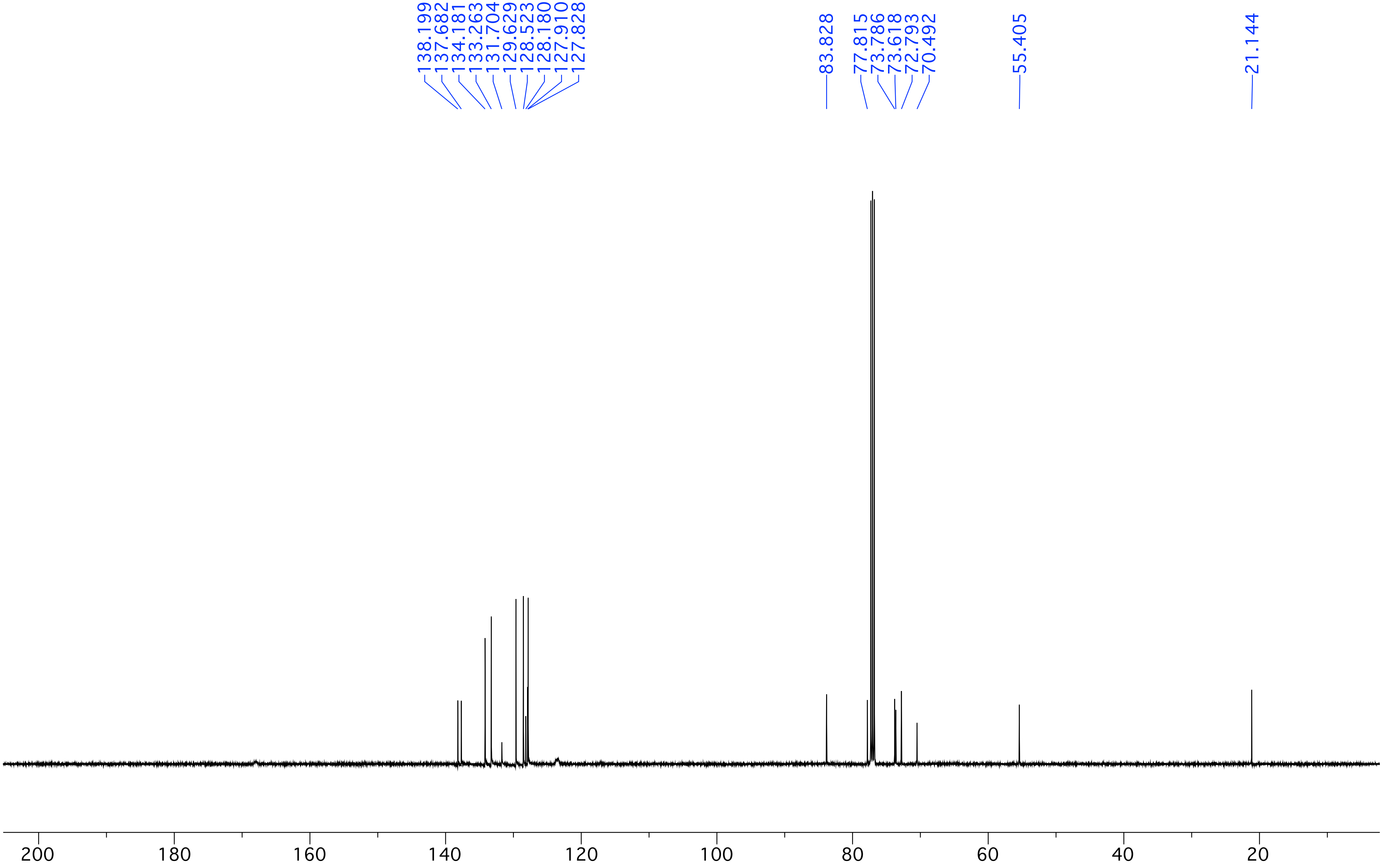
138.199
137.682
134.181
133.263
131.704
129.629
128.523
128.180
127.910
127.828
83.828
77.815
73.786
73.618
72.793
70.492
55.405
21.144
200
180
160
140
120
100
80
60
40
20

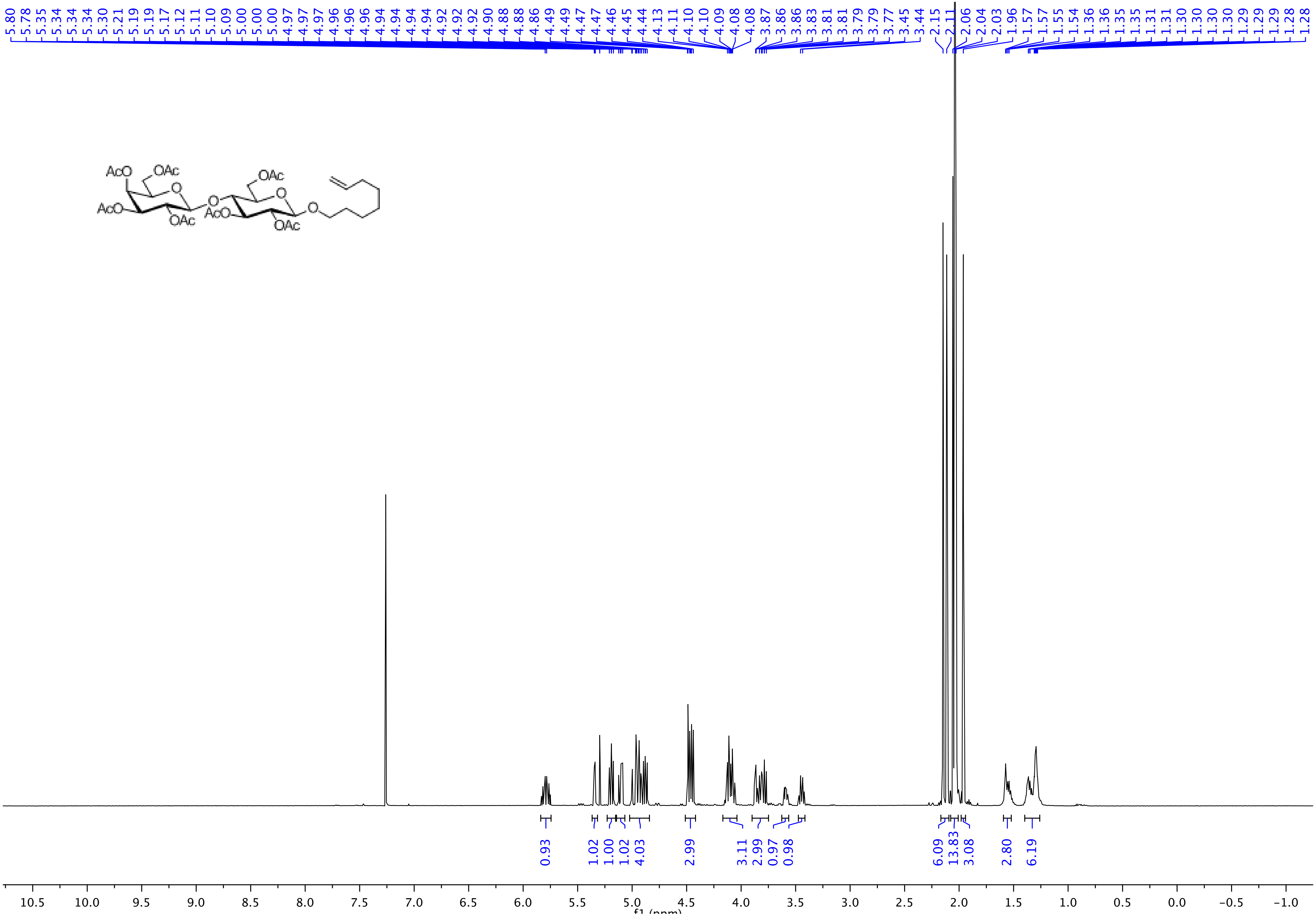
5.80 5.78 5.35 5.34 5.34 5.34 5.30 5.21 5.19 5.19 5.17 5.12 5.11 5.10 5.09 5.00 5.00 4.97 4.97 4.97 4.96 4.96 4.96 4.94 4.94 4.94 4.94 4.92 4.92 4.92 4.90 4.88 4.88 4.86 4.49 4.49 4.47 4.47 4.46 4.45 4.44 4.13 4.11 4.10 4.10 4.09 4.08 4.08 3.87 3.86 3.86 3.83 3.81 3.81 3.79 3.79 3.77 3.45 3.44 2.15 2.11 2.06 2.04 2.03 1.96 1.57 1.57 1.55 1.54 1.36 1.36 1.35 1.35 1.31 1.31 1.30 1.30 1.30 1.30 1.29 1.29 1.29 1.28 1.28
AcO
OAc
O
AcO
O
OAc
OAc
AcO
O
OAc
O
0.93
1.02
1.00
1.02
4.03
2.99
3.11
2.99
0.97
0.98
6.09
13.83
3.08
2.80
6.19
10.5 10.0 9.5 9.0 8.5 8.0 7.5 7.0 6.5 6.0 5.5 5.0 4.5 4.0 3.5 3.0 2.5 2.0 1.5 1.0 0.5 0.0 -0.5 -1.0
f1 (ppm

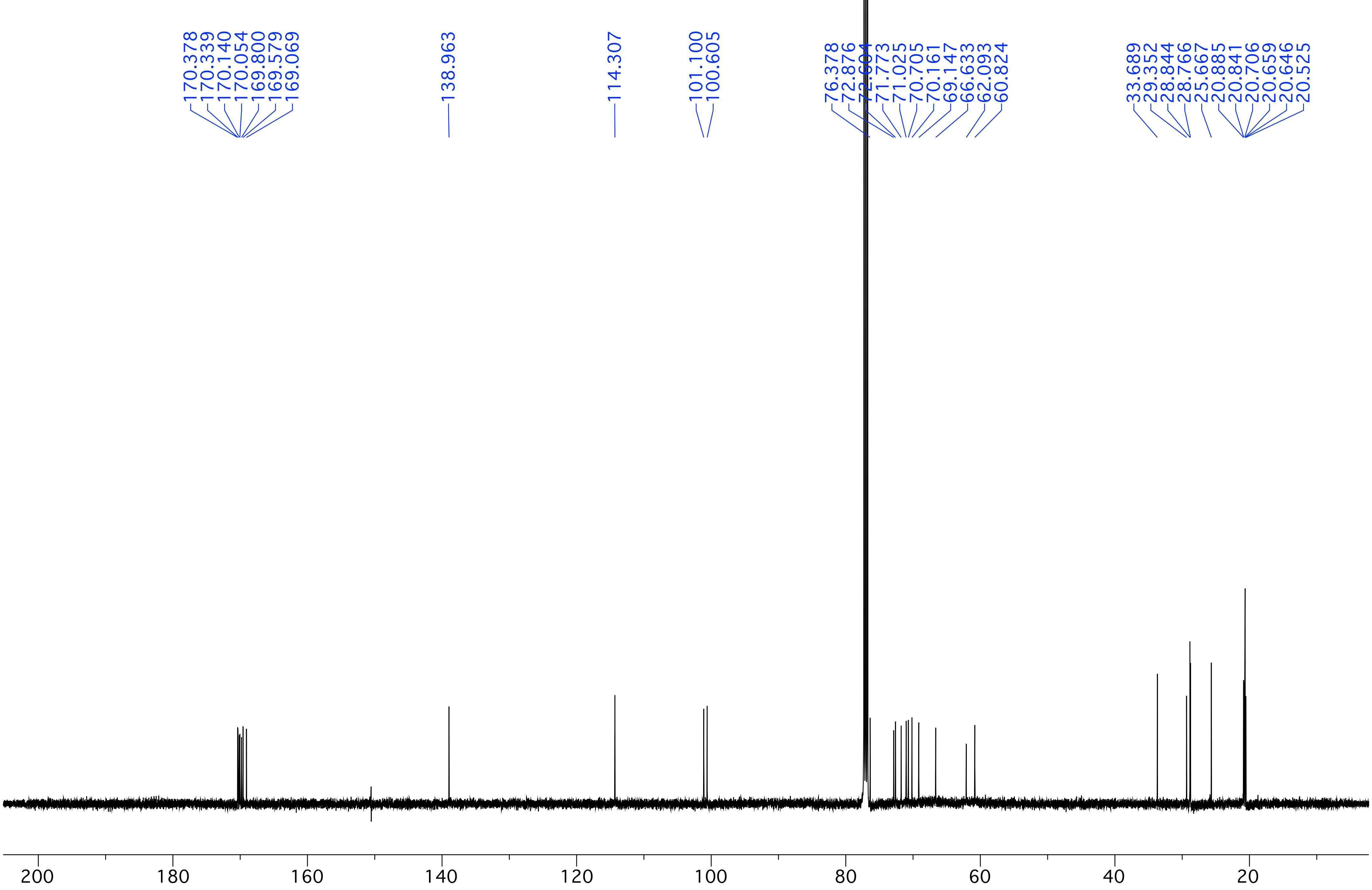

170.378
170.339
170.140
170.054
169.800
169.579
169.069
138.963
114.307
101.100
100.605
76.378
72.876
72.604
71.773
71.025
70.705
70.161
69.147
66.633
62.093
60.824
33.689
29.352
28.844
28.766
25.667
20.885
20.841
20.706
20.659
20.646
20.525
200
180
160
140
120
100
80
60
40
20

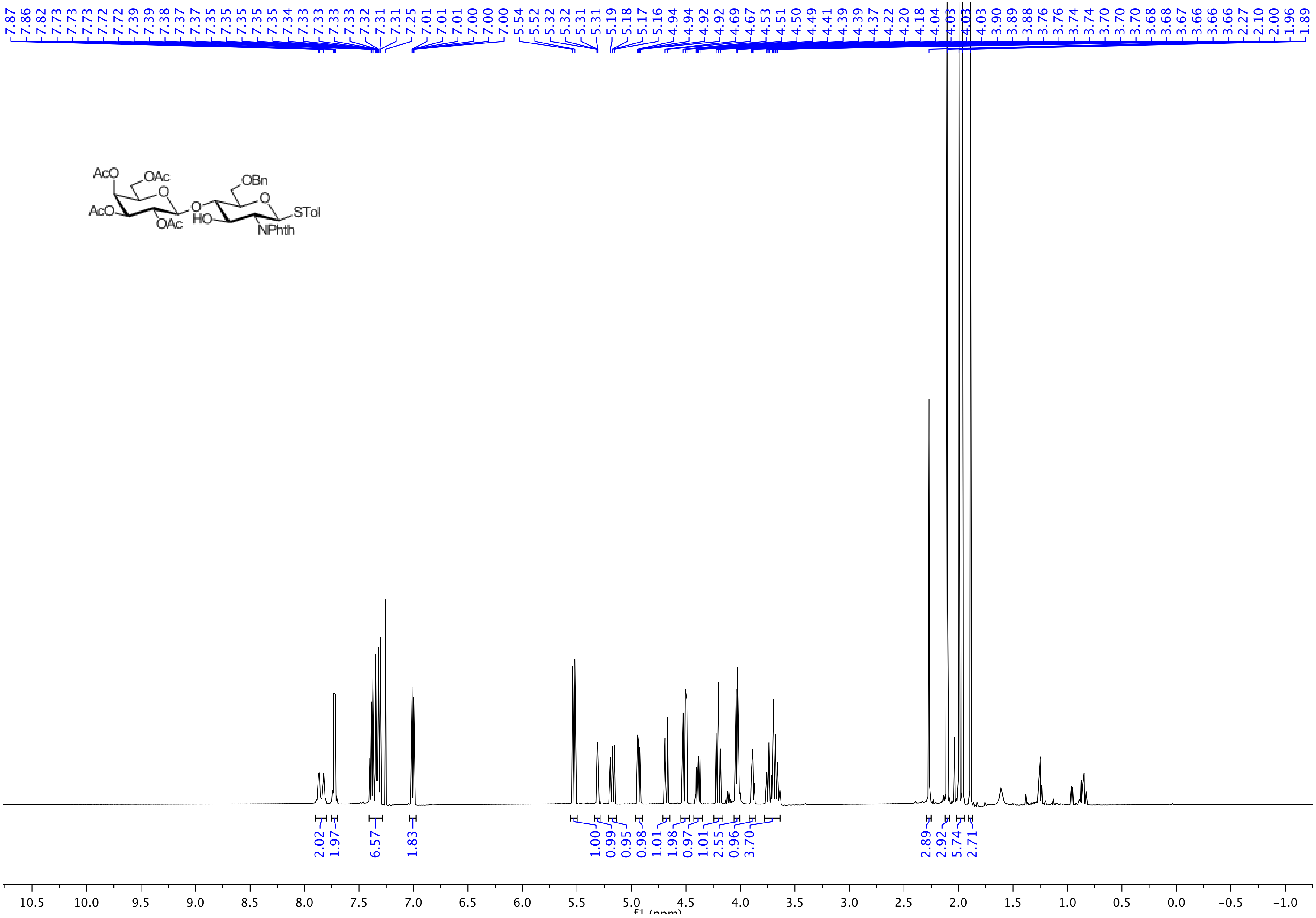

7.87 7.86 7.82 7.73 7.73 7.73 7.72 7.72 7.39 7.39 7.38 7.37 7.37 7.35 7.35 7.35 7.35 7.34 7.33 7.33 7.33 7.33 7.32 7.31 7.31 7.25 7.01 7.01 7.01 7.00 7.00 7.00 5.54 5.52 5.32 5.32 5.31 5.31 5.19 5.18 5.17 5.16 4.94 4.94 4.92 4.92 4.69 4.67 4.53 4.51 4.50 4.49 4.41 4.39 4.39 4.37 4.22 4.20 4.18 4.04 4.03 4.03 4.03 3.90 3.89 3.88 3.76 3.76 3.74 3.74 3.70 3.70 3.70 3.68 3.68 3.67 3.66 3.66 3.66 2.27 2.10 2.00 1.96 1.89
AcO
OAc
AcO
OAc
OBn
HO
STol
NPhth
2.02 1.97 6.57 1.83 1.00 0.99 0.95 0.98 1.01 1.98 0.97 1.01 2.55 0.96 3.70 2.89 2.92 5.74 2.71
10.5 10.0 9.5 9.0 8.5 8.0 7.5 7.0 6.5 6.0 5.5 5.0 4.5 4.0 3.5 3.0 2.5 2.0 1.5 1.0 0.5 0.0 -0.5 -1.0
f1 (ppm)

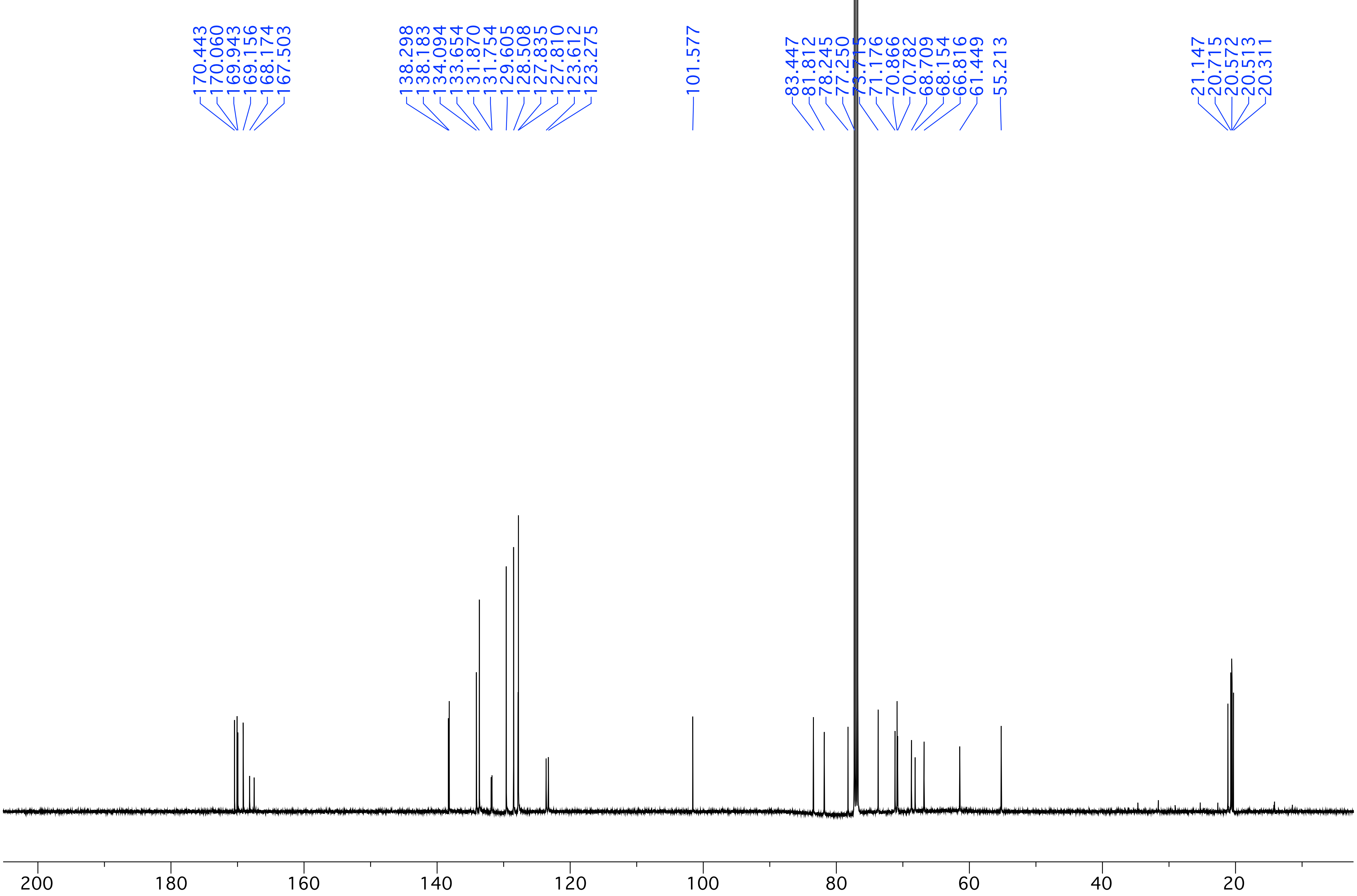
170.443
170.060
169.943
169.156
168.174
167.503
138.298
138.183
134.094
133.654
131.870
131.754
129.605
128.508
127.835
127.810
123.612
123.275
101.577
83.447
81.812
78.245
77.250
73.715
71.176
70.866
70.782
68.709
68.154
66.816
61.449
55.213
21.147
20.715
20.572
20.513
20.311
200
180
160
140
120
100
80
60
40
20

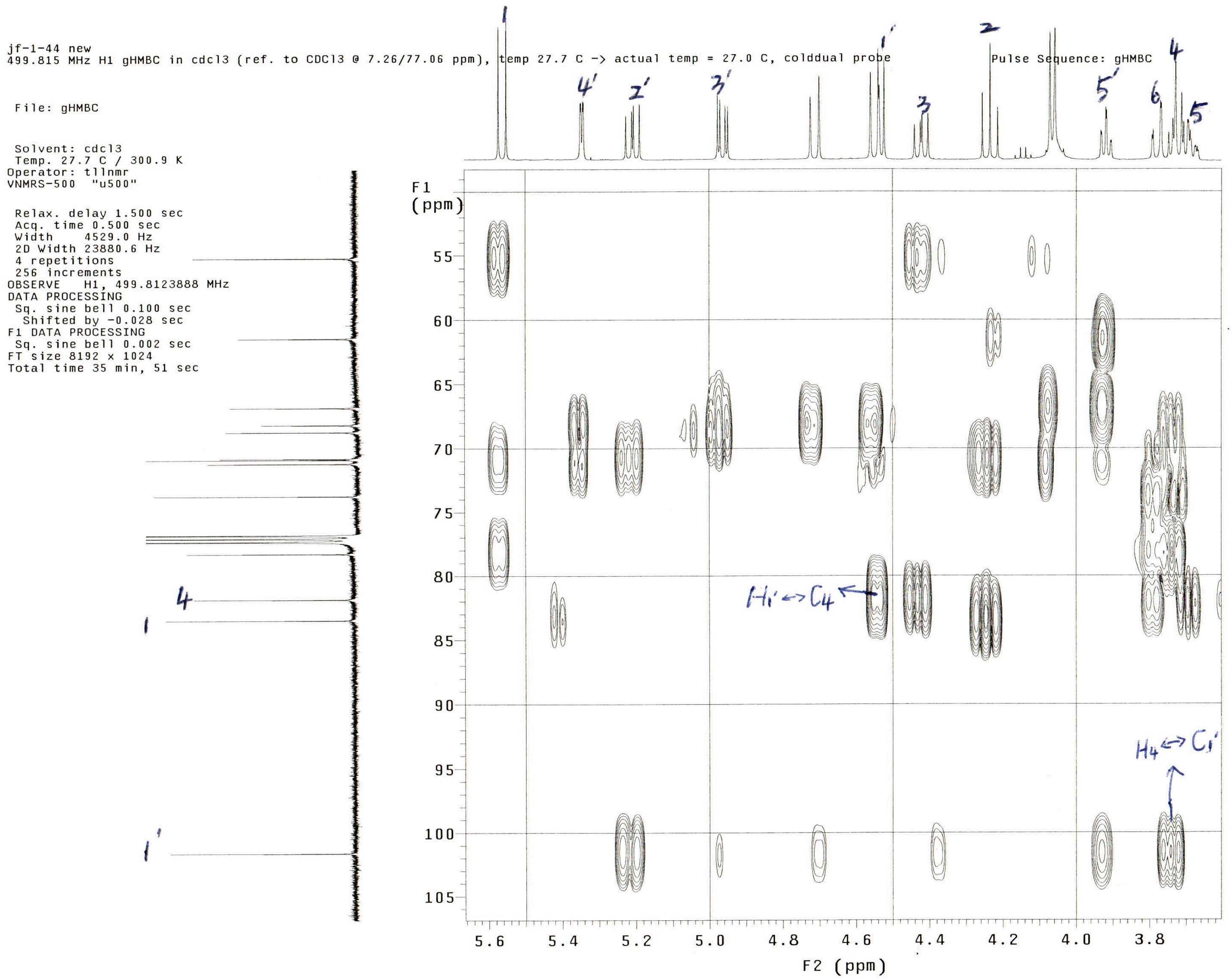
jf-1-44 new
499.815 MHz H1 gHMBC in cdcl3 (ref. to CDCl3 @ 7.26/77.06 ppm), temp 27.7 C -> actual temp = 27.0 C, colddual probe
Pulse Sequence: gHMBC
File: gHMBC
Solvent: cdcl3
Temp. 27.7 C / 300.9 K
Operator: tllnmr
VNMRS-500 "u500"
Relax. delay 1.500 sec
Acq. time 0.500 sec
Width 4529.0 Hz
2D Width 23880.6 Hz
4 repetitions
256 increments
OBSERVE H1, 499.8123888 MHz
DATA PROCESSING
Sq. sine bell 0.100 sec
Shifted by -0.028 sec
F1 DATA PROCESSING
Sq. sine bell 0.002 sec
FT size 8192 x 1024
Total time 35 min, 51 sec
F1 (ppm)
F2 (ppm)
H1' ↔ C4
H4 ↔ C1'

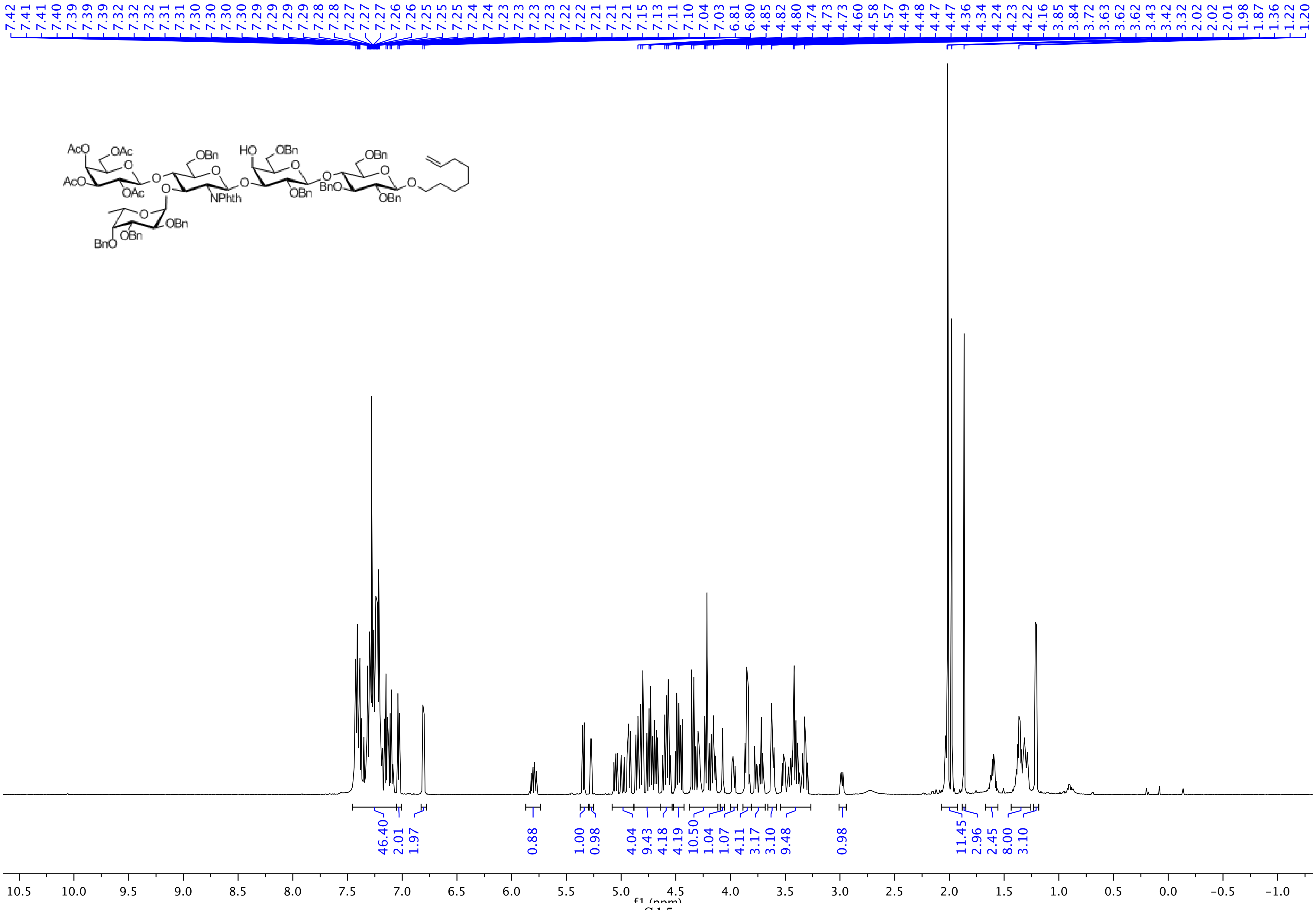
7.42 7.41 7.40 7.39 7.39 7.39 7.32 7.32 7.32 7.31 7.31 7.30 7.30 7.30 7.30 7.29 7.29 7.29 7.29 7.28 7.28 7.27 7.27 7.27 7.26 7.26 7.25 7.25 7.24 7.24 7.23 7.23 7.23 7.23 7.22 7.22 7.21 7.21 7.21 7.15 7.13 7.11 7.10 7.04 7.03 6.81 6.80 4.85 4.82 4.80 4.74 4.73 4.73 4.60 4.58 4.57 4.49 4.48 4.47 4.47 4.36 4.34 4.24 4.23 4.22 4.16 3.85 3.84 3.72 3.63 3.62 3.62 3.43 3.42 3.32 2.02 2.02 2.01 1.98 1.87 1.36 1.22 1.20
AcO
OAc
AcO
OAc
OBn
NPhth
HO
OBn
OBn
BnO
OBn
OBn
OBn
BnO
OBn
46.40 2.01 1.97 0.88 1.00 0.98 4.04 9.43 4.18 4.19 10.50 1.04 1.07 4.11 3.17 3.10 9.48 0.98 11.45 2.96 2.45 8.00 3.10
10.5 10.0 9.5 9.0 8.5 8.0 7.5 7.0 6.5 6.0 5.5 5.0 4.5 4.0 3.5 3.0 2.5 2.0 1.5 1.0 0.5 0.0 -0.5 -1.0
f1 (ppm)

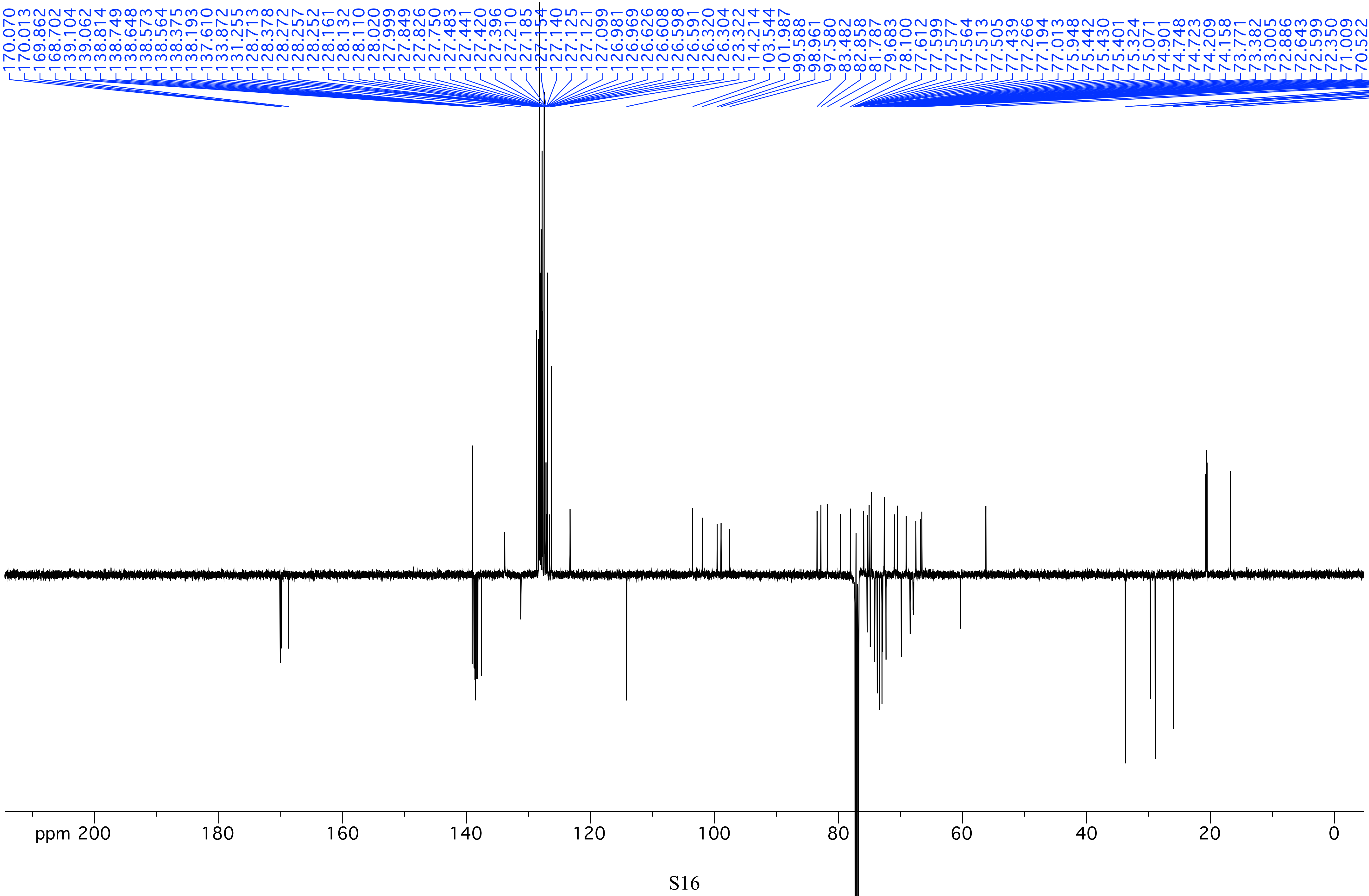

170.070
170.013
169.862
168.702
139.104
139.062
138.814
138.749
138.648
138.573
138.564
138.375
138.193
137.610
133.872
131.255
128.713
128.378
128.272
128.257
128.252
128.161
128.132
128.110
128.020
127.999
127.849
127.826
127.750
127.483
127.441
127.420
127.396
127.210
127.185
127.154
127.140
127.125
127.121
127.099
126.981
126.969
126.626
126.608
126.598
126.591
126.320
126.304
123.322
114.214
103.544
101.987
99.588
98.961
97.580
83.482
82.858
81.787
79.683
78.100
77.612
77.599
77.577
77.564
77.513
77.505
77.439
77.266
77.194
77.013
75.948
75.442
75.430
75.401
75.324
75.071
74.901
74.748
74.723
74.209
74.158
73.771
73.382
73.005
72.886
72.643
72.599
72.350
71.009
70.522
ppm 200
180
160
140
120
100
80
60
40
20
0

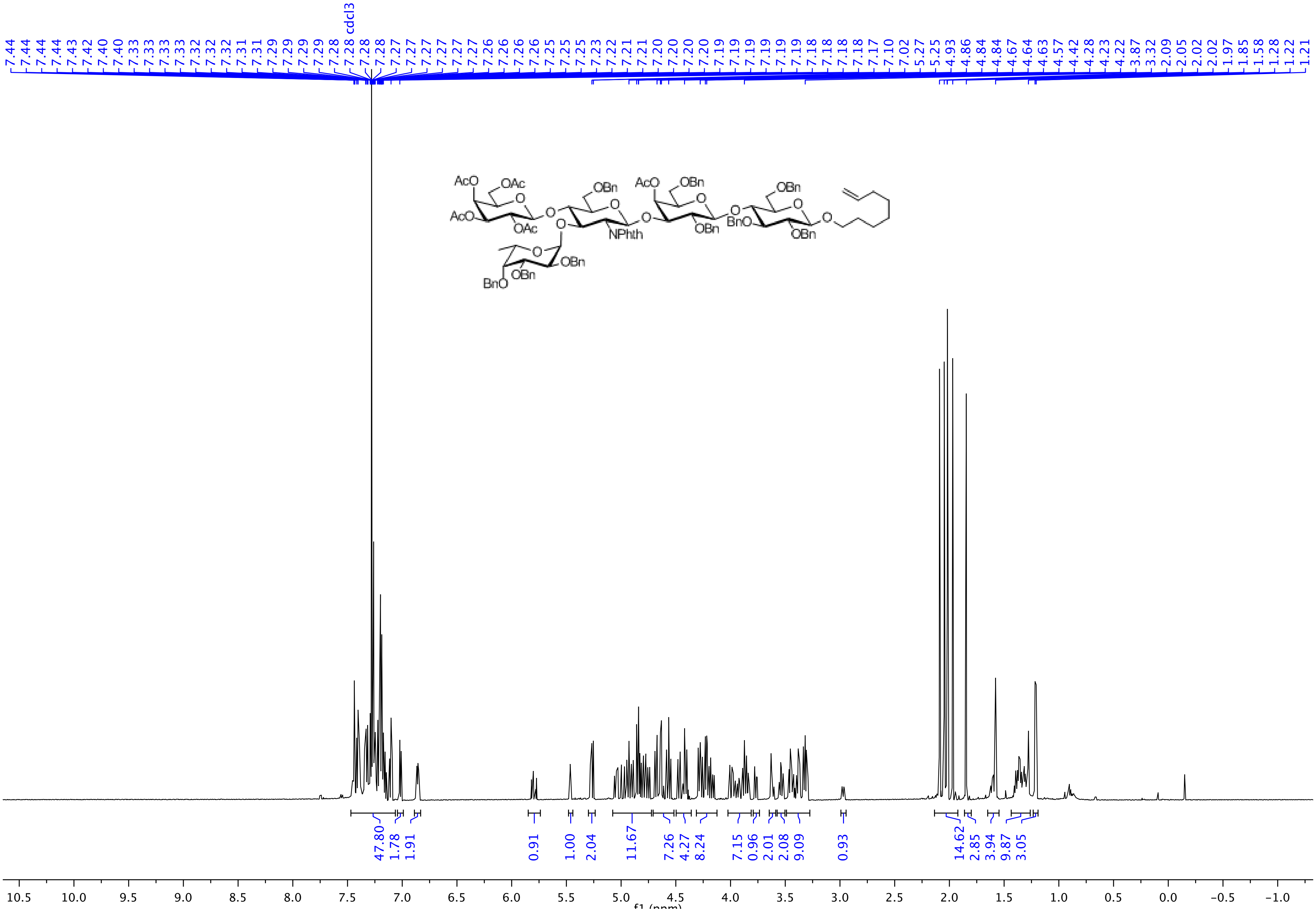
7.44 7.44 7.44 7.43 7.42 7.40 7.40 7.33 7.33 7.33 7.33 7.32 7.32 7.31 7.31 7.29 7.29 7.29 7.29 7.28 7.28 cdcl3 7.28 7.28 7.27 7.27 7.27 7.27 7.26 7.26 7.26 7.25 7.25 7.25 7.23 7.22 7.21 7.21 7.20 7.20 7.20 7.19 7.19 7.19 7.19 7.19 7.18 7.18 7.18 7.17 7.10 7.02 5.27 5.25 4.93 4.86 4.84 4.84 4.67 4.64 4.63 4.57 4.42 4.28 4.23 4.22 3.87 3.32 2.09 2.05 2.02 2.02 1.97 1.85 1.58 1.28 1.22 1.21
AcO
OAc
AcO
OAc
OBn
NPhth
OBn
BnO
OBn
OBn
OBn
BnO
47.80 1.78 1.91 0.91 1.00 2.04 11.67 7.26 4.27 8.24 7.15 0.96 2.01 2.08 9.09 0.93 14.62 2.85 3.94 9.87 3.05
10.5 10.0 9.5 9.0 8.5 8.0 7.5 7.0 6.5 6.0 5.5 5.0 4.5 4.0 3.5 3.0 2.5 2.0 1.5 1.0 0.5 0.0 -0.5 -1.0
f1 (ppm)

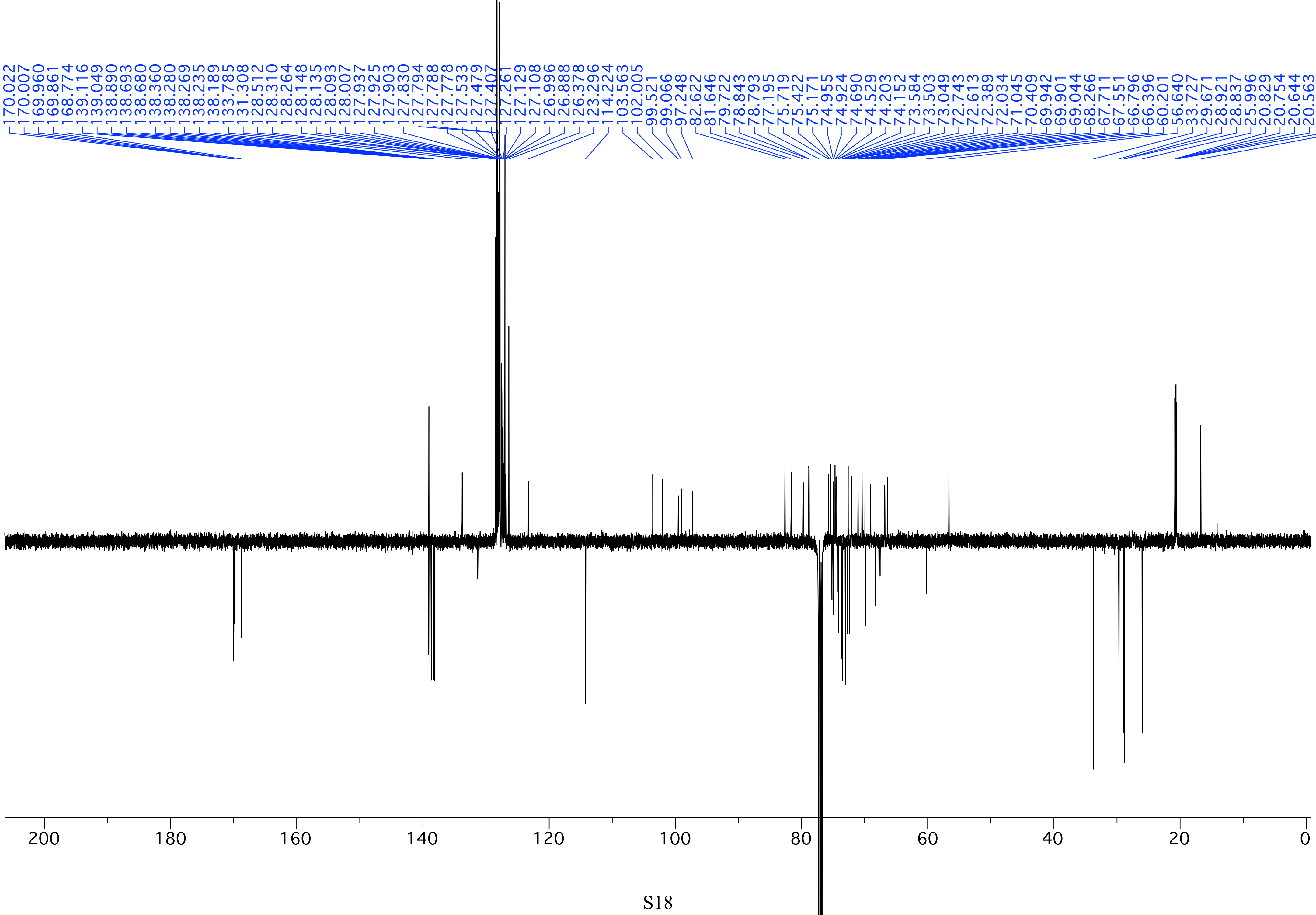
170.022
170.007
169.960
169.861
168.774
139.116
139.049
138.890
138.693
138.680
138.360
138.280
138.269
138.235
138.189
133.785
131.308
128.512
128.310
128.264
128.148
128.135
128.093
128.007
127.937
127.925
127.903
127.830
127.794
127.788
127.778
127.533
127.479
127.407
127.261
127.129
127.108
126.996
126.888
126.378
123.296
114.224
103.563
102.005
99.521
99.066
97.248
82.622
81.646
79.722
78.843
78.793
77.195
75.719
75.422
75.171
74.955
74.924
74.690
74.529
74.203
74.152
73.584
73.503
73.049
72.743
72.613
72.389
72.034
71.045
70.409
69.942
69.901
69.044
68.266
67.711
67.551
66.796
66.396
60.201
56.640
33.727
29.671
28.921
28.837
25.996
20.829
20.754
20.644
20.563
200
180
160
140
120
100
80
60
40
20
0

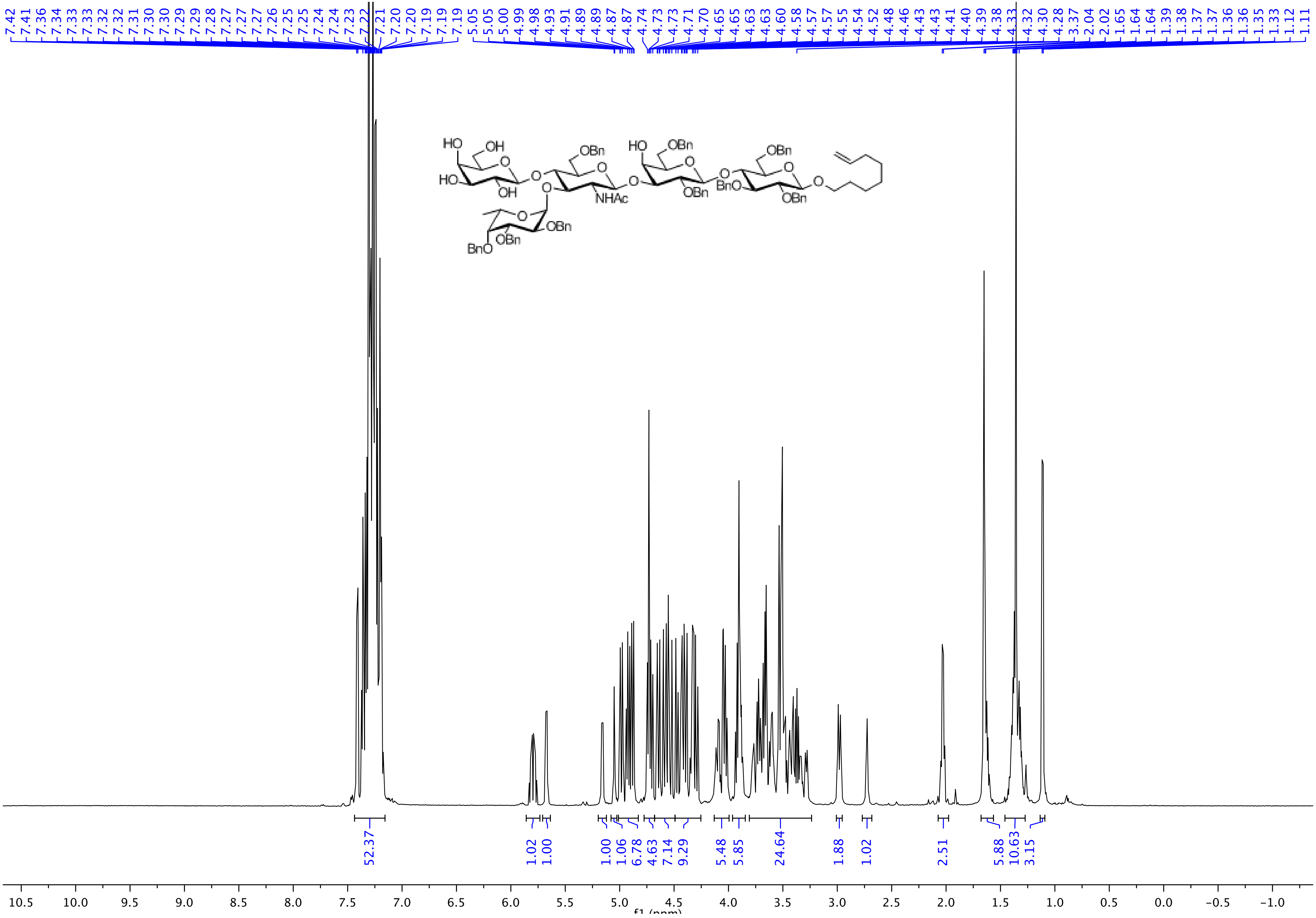
7.42 7.41 7.36 7.34 7.33 7.33 7.32 7.32 7.31 7.30 7.30 7.29 7.29 7.28 7.27 7.27 7.26 7.25 7.25 7.24 7.24 7.23 7.22 7.21 7.20 7.20 7.19 7.19 5.05 5.05 5.00 4.99 4.98 4.93 4.91 4.89 4.89 4.87 4.87 4.74 4.73 4.73 4.71 4.70 4.65 4.65 4.63 4.63 4.60 4.58 4.57 4.57 4.55 4.54 4.52 4.48 4.46 4.43 4.43 4.41 4.40 4.39 4.38 4.33 4.32 4.30 4.28 3.37 2.04 2.02 1.65 1.64 1.64 1.39 1.38 1.37 1.37 1.36 1.36 1.35 1.33 1.12 1.11
52.37
1.02
1.00
1.00
1.06
6.78
4.63
7.14
9.29
5.48
5.85
24.64
1.88
1.02
2.51
5.88
10.63
3.15
10.5 10.0 9.5 9.0 8.5 8.0 7.5 7.0 6.5 6.0 5.5 5.0 4.5 4.0 3.5 3.0 2.5 2.0 1.5 1.0 0.5 0.0 -0.5 -1.0

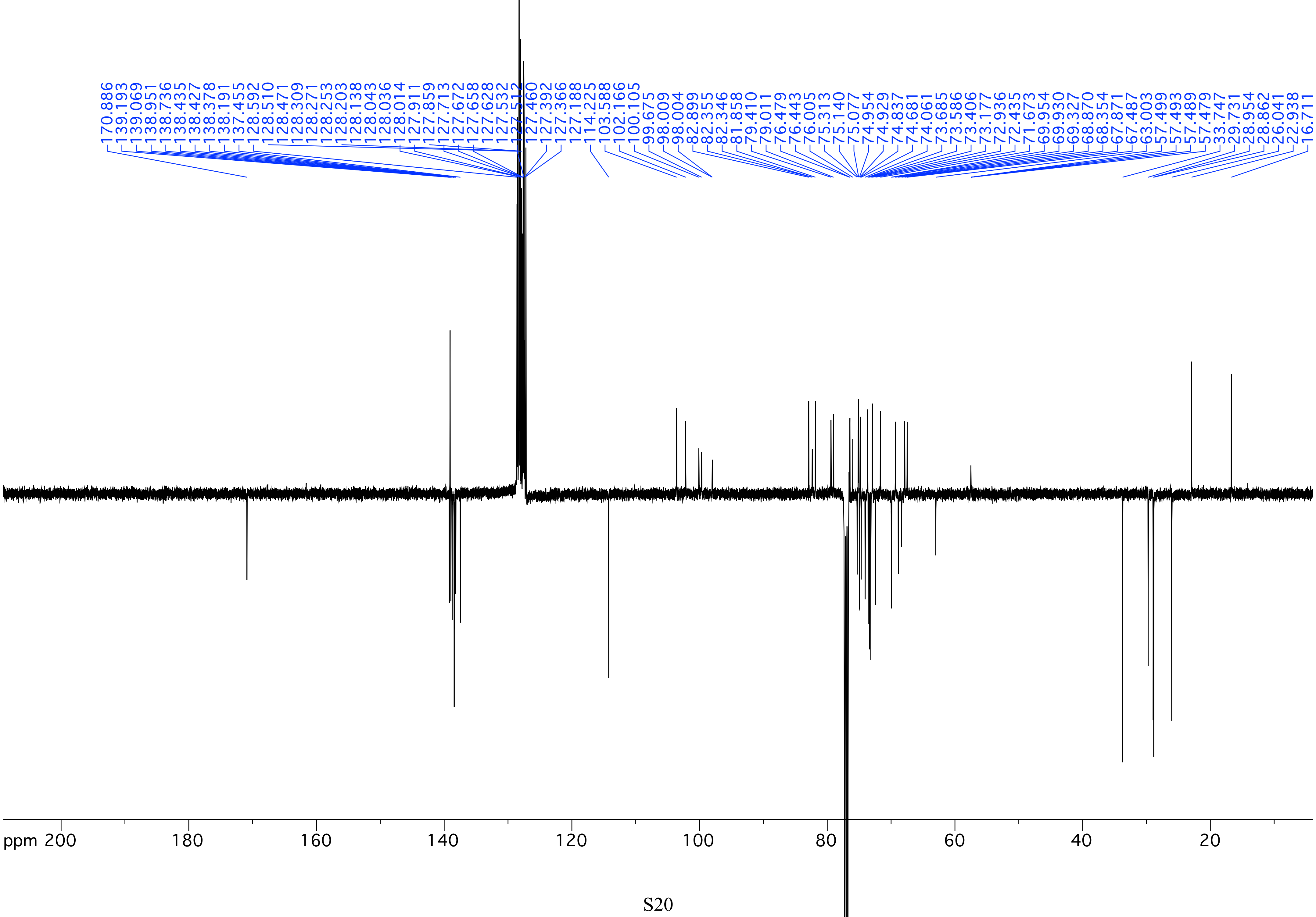

170.886
139.193
139.069
138.951
138.736
138.435
138.427
138.378
138.191
137.455
128.592
128.510
128.471
128.309
128.271
128.253
128.203
128.138
128.043
128.036
128.014
127.911
127.859
127.713
127.672
127.658
127.628
127.532
127.512
127.460
127.392
127.366
127.188
114.225
103.588
102.166
100.105
99.675
98.009
98.004
82.899
82.355
82.346
81.858
79.410
79.011
76.479
76.443
76.005
75.313
75.140
75.077
74.954
74.929
74.837
74.681
74.061
73.685
73.586
73.406
73.177
72.936
72.435
71.673
69.954
69.930
69.327
68.870
68.354
67.871
67.487
63.003
57.499
57.493
57.489
57.479
33.747
29.731
28.954
28.862
26.041
22.938
16.711
ppm 200
180
160
140
120
100
80
60
40
20

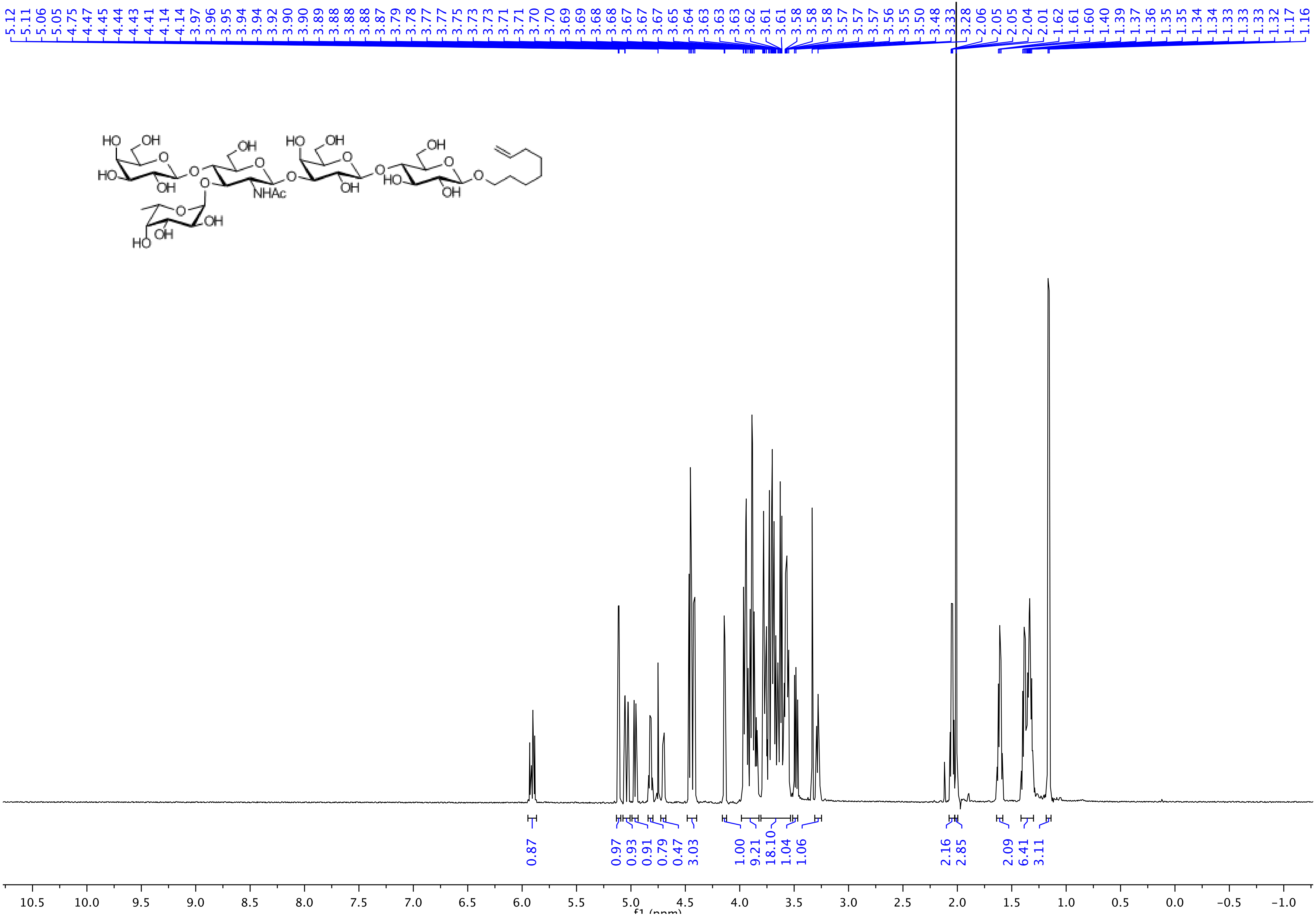

5.12
5.11
5.06
5.05
4.75
4.47
4.45
4.44
4.43
4.41
4.14
4.14
3.97
3.96
3.95
3.94
3.94
3.92
3.90
3.90
3.89
3.88
3.88
3.88
3.87
3.79
3.78
3.77
3.77
3.75
3.73
3.73
3.71
3.71
3.70
3.70
3.69
3.69
3.68
3.68
3.67
3.67
3.67
3.65
3.64
3.63
3.63
3.62
3.61
3.61
3.58
3.58
3.58
3.57
3.57
3.57
3.56
3.55
3.50
3.48
3.33
3.28
2.06
2.05
2.05
2.04
2.01
1.62
1.61
1.60
1.40
1.39
1.37
1.36
1.35
1.35
1.34
1.34
1.33
1.33
1.33
1.32
1.17
1.16
HO
OH
O
NHAc
0.87
0.97
0.93
0.91
0.79
0.47
3.03
1.00
9.21
18.10
1.04
1.06
2.16
2.85
2.09
6.41
3.11
10.5
10.0
9.5
9.0
8.5
8.0
7.5
7.0
6.5
6.0
5.5
5.0
4.5
4.0
3.5
3.0
2.5
2.0
1.5
1.0
0.5
0.0
-0.5
-1.0
f1 (ppm)

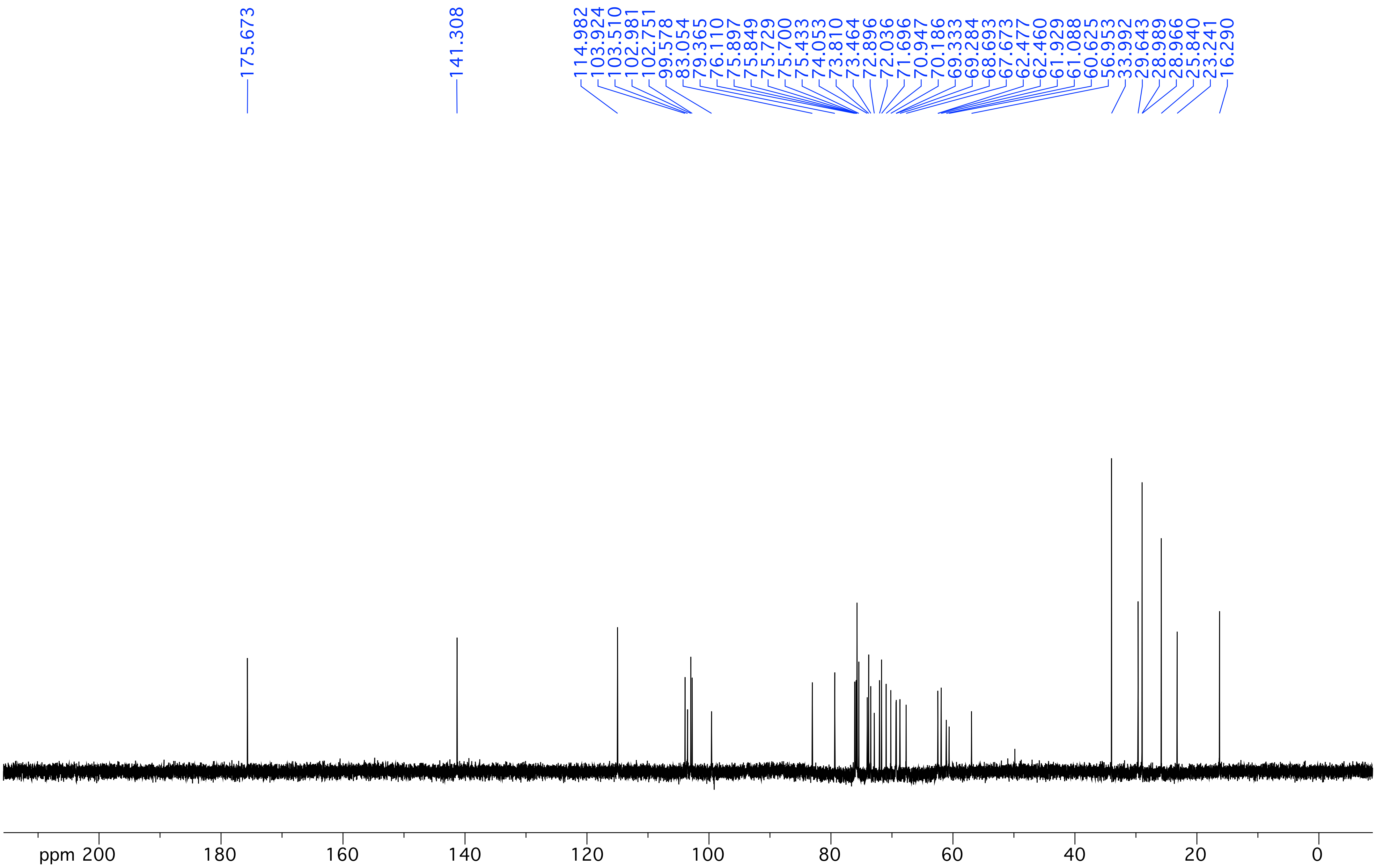
175.673
141.308
114.982
103.924
103.510
102.981
102.751
99.578
83.054
79.365
76.110
75.897
75.849
75.729
75.700
75.433
74.053
73.810
73.464
72.896
72.036
71.696
70.947
70.186
69.333
69.284
68.693
67.673
62.477
62.460
61.929
61.088
60.625
56.953
33.992
29.643
28.989
28.966
25.840
23.241
16.290
ppm 200
180
160
140
120
100
80
60
40
20
0

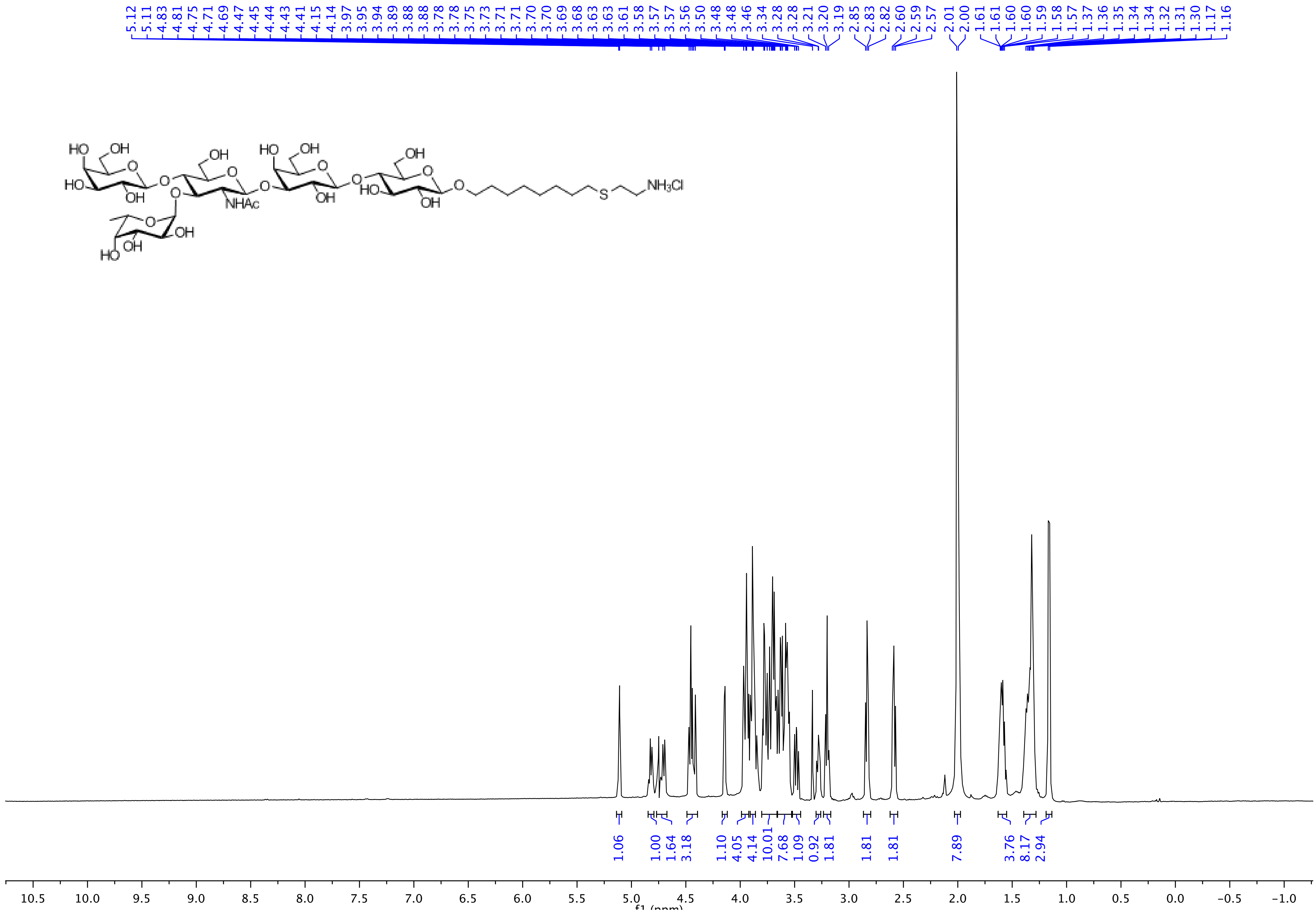

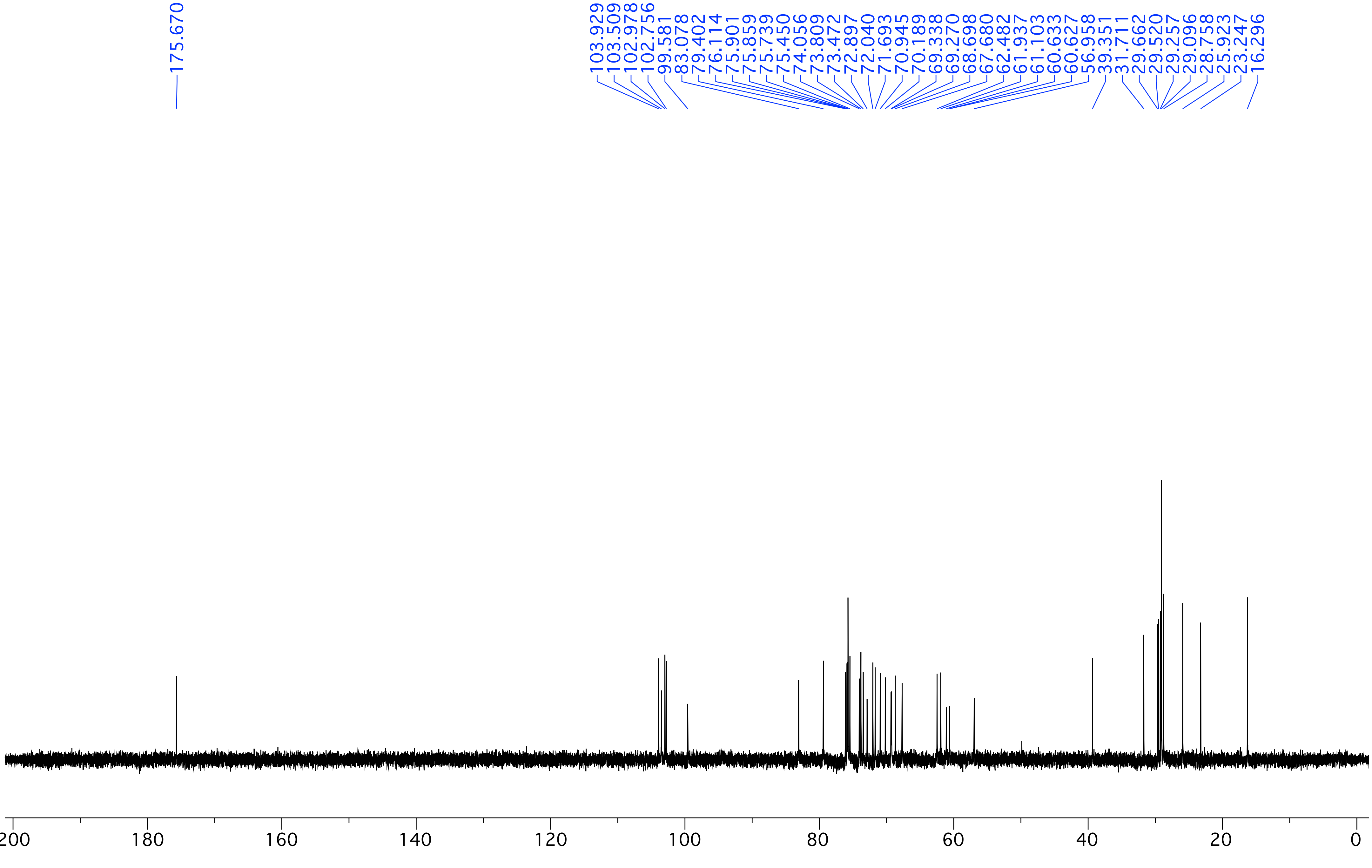
175.670
103.929
103.509
102.978
102.756
99.581
83.078
79.402
76.114
75.901
75.859
75.739
75.450
74.056
73.809
73.472
72.897
72.040
71.693
70.945
70.189
69.338
69.270
68.698
67.680
62.482
61.937
61.103
60.633
60.627
56.958
39.351
31.711
29.662
29.520
29.257
29.096
28.758
25.923
23.247
16.296
200
180
160
140
120
100
80
60
40
20
0

D:\Data\MSService\2013\201304\130425_LZou_Lowary\LZ_05093_HSA\0_E7\1\1SLin

Comment 1 LZ_05093_HSA, SA, LP_20-100_kDa, AC800
Comment 2 L.Zou, T. Lowary

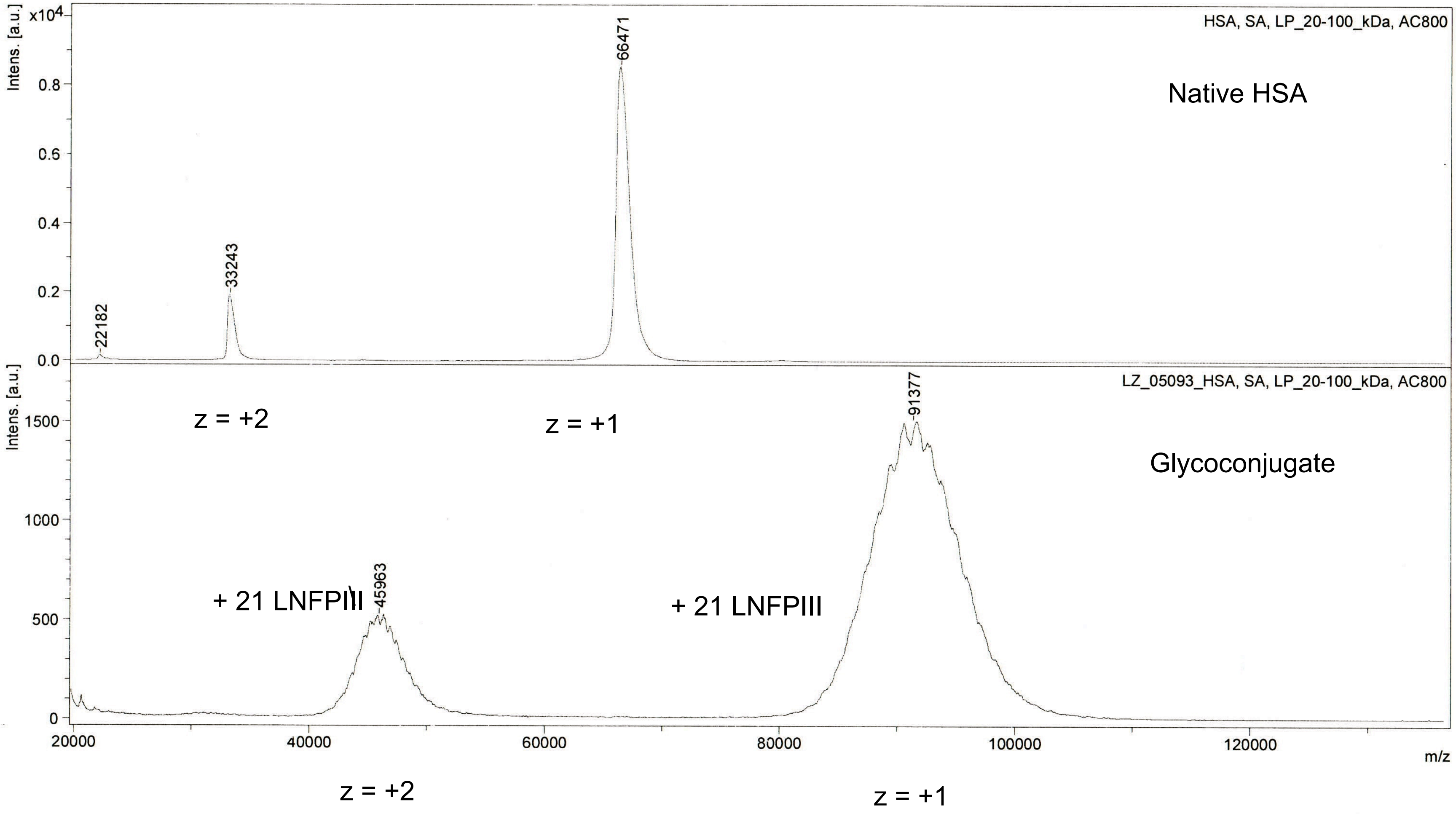

Bruker Daltonics flexAnalysis printed: 4/25/2013 9:42:47 AM
